# Unraveling the multiplicity of geranylgeranyl reductases in Archaea: potential roles in saturation of terpenoids

**DOI:** 10.1007/s00792-023-01330-2

**Published:** 2024-01-27

**Authors:** Alka Rao, Arnold J. M. Driessen

**Affiliations:** Department of Molecular Microbiology, Groningen Biomolecular Science and Biotechnology Institute, University of Groningen, 9747 AG Groningen, The Netherlands

**Keywords:** Terpenoids, GGR paralogs, Archaea, Saturation, GDGT, Isoprenoids, Extremophiles

## Abstract

The enzymology of the key steps in the archaeal phospholipid biosynthetic pathway has been elucidated in recent years. In contrast, the complete biosynthetic pathways for proposed membrane regulators consisting of polyterpenes, such as carotenoids, respiratory quinones, and polyprenols remain unknown. Notably, the multiplicity of geranylgeranyl reductases (GGRs) in archaeal genomes has been correlated with the saturation of polyterpenes. Although GGRs, which are responsible for saturation of the isoprene chains of phospholipids, have been identified and studied in detail, there is little information regarding the structure and function of the paralogs. Here, we discuss the diversity of archaeal membrane-associated polyterpenes which is correlated with the genomic loci, structural and sequence-based analyses of GGR paralogs.

## Introduction

In 1977, the Woesian revolution recognized archaea as a distinct domain of life (Balch et al. 1977). Archaeal membranes have been studied comprehensively for their intriguing chemical composition, as well as for their contribution to robustness when thriving in extreme environments. A hallmark of archaeal phospholipids is the presence of ether bonds that connect the sn-glycerol-1-phosphate (G1P) backbone to two isoprenoid chains (Jain et al. 2014a, b). It differs from the phospholipids in bacterial membranes, which consist of a sn-glycerol-3-phosphate (G3P) backbone with ester bonded to two fatty acyl chains (Jain et al. 2014a, b). This inherent difference in chemical composition of archaeal and bacterial membranes has been linked to their early evolution and is termed as the ‘lipid divide’, reflecting the split of the last universal common ancestor (LUCA) into these two domains that occurred over 4.3 billion years ago. This ‘lipid divide’ is still evident in the organisms that live today, where the bulk phospholipid composition of most organisms adheres to the ‘lipid divide’ conundrum.

It has been proposed that mixed heterochiral membranes are intrinsically unstable, and this would have been the driving force of the ‘lipid divide’ where life originated from the last universal common ancestor, LUCA (Koga et al. 1998; Wächtershäuser 2003). This hypothesis has been challenged by in vitro studies showing that liposomes composed of mixed heterochiral membranes are stable (Haruo Shimada and Yamagishi 2011). Furthermore, reconstruction of the archaeal phospholipid biosynthetic pathway in *E. coli* resulted in mixed heterochiral membranes that supported the growth of this engineered strain (Caforio et al. 2018). Additionally, the reconstruction of lipid biosynthetic pathways from uncultured *Lokiarchaeota*_BC1 and *Thorarchaeota* AB led to the production of a heterochiral mixed membrane in *Saccharomyces cerevisiae*, wherein the G3P-based enantiomer constituted a minor fraction (0.51–0.69%) of the total unsaturated archaeol pool (Zhang et al. 2023). In-vitro studies have showed that the addition of isoprenoids enhances the stability of fatty acid vesicles across a pH range and under various concentrations of ionic species (Jordan et al. 2019). Hence, membrane instability is unlikely the main reason for the ‘lipid divide’, and rather, it is believed that LUCA was not a single life form, but a heterogeneous mixture of organisms which eventually led to the successful emergence of two lineages—Archaea and Bacteria. Hence, the question about the driving force behind the ‘lipid divide’ remains unanswered for now. Recently, it was hypothesized that archaeal and bacterial membranes are differently optimized for efficient energy transduction, where in archaeal membranes would be optimized for limited H^+^ leaks under a wide range of environmental conditions whereas bacterial membranes would be optimized for efficient lateral H^+^ transport along the carbonyl oxygen of the ester bond supporting localized chemiosmosis (Mencía 2020).

Likely due to horizontal or convergent evolution, exceptions to this divide also seem to exist. For instance, members of *Ca.*Cloacimonetes from the bacterial Fibrobacteres–Chlorobi–Bacteroidetes (FCB) superphylum have the potential to synthesize archaeal ether-linked phospholipids with a presumed G1P stereochemistry (Villanueva et al. 2021). Additionally, bacteria from Rubrobacterales contain ~ 46% ether-linked phospholipids of their core lipids (Sinninghe Damsté et al. 2023). A similar phenomenon is observed in the hyperthermophilic bacterium *Thermotoga maritima* wherein mixed membrane-spanning tetraethers/tetraesters are found (Sahonero-Canavesi et al. 2022). However, in both these studies, the stereochemistry of the phospholipids was not determined.

**Fig. 1:**
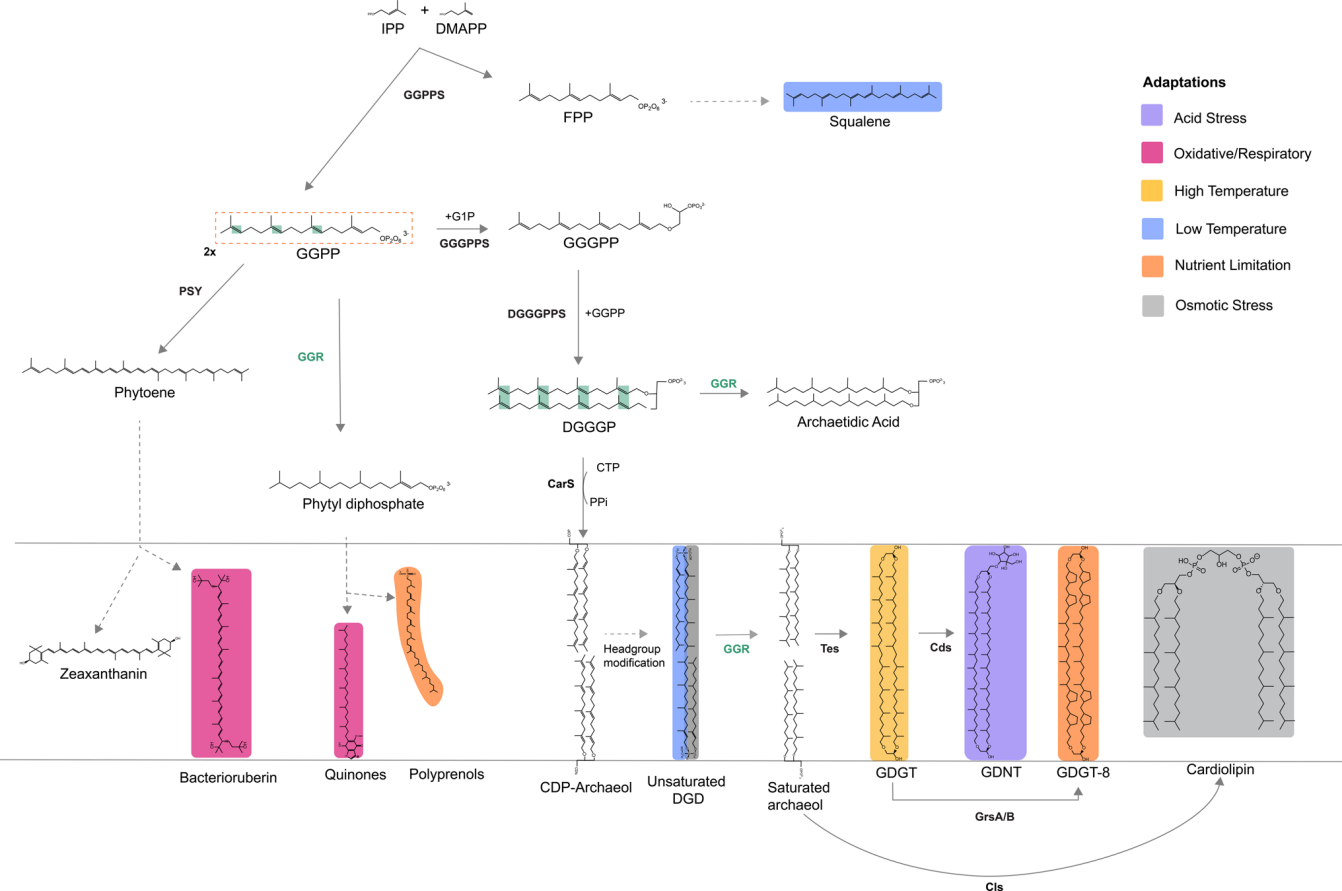
Schematic representation of the archaeal lipid biosynthesis pathway: colors represent the membrane adaptations discussed in this study. Dotted lines indicate a multiple-step pathway that has not yet been characterized in archaea. The green blocks represent the saturation sites of the GGR. This figure was adapted with permission from (Salvador-Castell et al. 2019)

In recent years, the biosynthetic pathway of archaeal membrane phospholipids has been elucidated near to completion (Jain et al. 2014a, b; Lloyd et al. 2022). This pathway is initiated with two isoprenoid building blocks, dimethylallyl pyrophosphate (DMAPP) and isopentenyl pyrophosphate (IPP), biosynthesized via the alternate or classical mevalonate pathway (Fig. 1) (Rastädter et al. 2020a, b). Geranylgeranyl pyrophosphate synthase (GGPPS) catalyzes the condensation of IPP and DMAPP to form geranylgeranyl pyrophosphate (GGPP) or farnesyl pyrophosphate (FPP) (Fig. 1) (Jain et al. 2014a, b; Chen and Poulter 1993). GGPP is condensed with the G1P backbone by geranylgeranyl glycerol phosphate synthase (GGGPS) to form GGGP (Fig. 1) (Jain et al. 2014a, b). GGGP is processed by digeranylgeranyl glyceryl phosphate synthase (DGGGPS) to form DGGGP (Ren et al. 2020). Next, DGGGP is activated by CarS with CTP for polar headgroup attachment, yielding CMP-DGGGP or CDP archaeols (Jain et al. 2014a, b). CDP-archaeol is a key intermediate in this pathway and a precursor for polar headgroup diversification (Jain et al. 2014a, b). There is uncertainty at what stage the isoprenoid chains are saturated by geranylgeranyl reductase (GGR) as saturated archaetidic acid is a poor substrate for CarS (Jain et al. 2014a, b). However, GGR can reduce DGGGP to archaetidic acid in vitro (Sato et al. 2008). Next, polar head group differentiation occurs that involves members of the universal family of transferases (Jain et al. 2014a, b).

Membrane-spanning glycerol dialkyl glycerol tetrathers (GDGTs) are formed through tail-to-tail condensation of saturated archaeols or dialkyl glycerol diethers (DGDs) via tetraether lipid synthase (Tes, radical SAM enzyme) (Lloyd et al. 2022; Zeng et al. 2022). GDGTs can be linked to the calditol headgroup forming glycerol dialkyl nonnitol tetraether (GDNTs) through another rSAM enzyme, calditol synthase (Cds) (Zeng et al. 2018). GDGTs can be found in nature with cyclopentane rings ranging from 0 to 8 (Sinninghe Damsté et al. 2002). Cyclized GDGTs increase the membrane packing and stability (Zhou et al. 2019). The GDGT ring synthases—GrsA and GrsB—introduce rings at the C-7 and C-3 positions, respectively, in the GDGT core lipid (Zeng et al. 2019), whereas the enzyme that introduces a hexyl ring in the tetraether lipids of Thaumarcheota has not been identified yet. The incorporation of these rings in archaeal GDGTs is regulated by pH, temperature, energy availability, and electron donor flux (Zhou et al. 2019; Yang et al. 2022). It is also a diagnostic tool to identify different clades of archaea. Additionally, GDGTs can be modified by the addition of an extra-cross linkage between two isoprenoid chains to form H-shaped GDGTs or glycerol monoalkyl glycerol tetraethers (GMGT) (Li et al. 2023). This linkage is catalyzed by the rSAM enzyme H-GDGT bridge synthase (Hbs) (Li et al. 2023). Remarkably, the distribution of Hbs is restricted to obligate anerobic archaeal genomes and metagenomes samples from anoxic environments (Li et al. 2023).

Apart from membrane lipids, isoprene or terpene units can undergo chain elongation and modifications (like cyclization) to form respiratory quinones or carotenoids or polyprenols in archaea. As a deviation of the aforementioned phospholipid biosynthetic pathway, GGPP can also undergo partial reduction by GGR to form phytyl diphosphate, which for instance in *Sulfolobus acidocaldarius* is a precursor for caldariellaquinones (CQ) (Sasaki et al. 2011). Additionally, two molecules of GGPP can condense to form phytoene via phytoene synthase (PSY) (Fig. 1) (Giani et al. 2020; De Castro et al. 2022). The biosynthesis of phytoene is a crucial regulatory step in carotenogenesis, which subsequently leads to the formation of lycopene or zeaxanthanin (De Castro et al. 2022). The enzymes responsible for chain elongation and modification of membrane-associated terpenoids in archaea remain unknown. Based on the canonical pathways in bacteria, this likely occurs through *cis*-isoprenyl diphosphate synthase (IPPS) or *trans*-IPPS enzymes which catalyze the head-to-tail condensation of isoprene units (Hoshino and Villanueva 2023). Specifically, the synthesis of squalene and carotenoids in archaea could be catalyzed by homologs of enzymes, such as phytoene synthase (CrtB) or squalene synthase (SQS) (Hoshino and Villanueva 2023).

## Saturation in archaeal membranes

Some Archaea thrive in extreme and unstable environments of low or high temperatures (as low as 0 °C, or up to 121 °C), acidic or alkaline pH (as low as pH 0.8, or up to pH 11), salinity (saturated salt lakes with a_w_ as low as 0.6), and pressure (up to 1100 bar) (Baker et al. 2020). Some are even polyextremophiles which inhabit environments with multiple extremes. Further, not all archaea are extremophilic and this domain also contains mesophiles that thrive in more moderate environments. The physicochemical properties of archaeal membranes are distinctive and modulated through unique mechanisms (Yosuke Koga 2012). However, different environments require distinct adaptations to the membranes; hence, various mechanisms of membrane adaptation can be found. Studies on the ion and proton permeability of diether-based archaeal liposomes revealed the presence of methyl groups on the phospholipid tails, and the ether bond present between the glycerol backbone and hydrophobic tail aids in the low permeability of membranes formed from diether phospholipids (Łapińska et al. 2023). However, liposomes consisting of only diether G1P-based archaeal lipids show a higher proton permeability compared to liposomes composed mostly of tetraether lipids from *S. acidocaldarius* (Łapińska et al. 2023; Komatsu and Chong 1998; Gmajner et al. 2011; Chong et al. 2017). Furthermore, the membranes of archaea may harbor various proportions of DGDs and GDGTs, which helps in regulating the permeability characteristics depending on the environmental conditions. For example, hyperthermophilic methanogens increase the membrane-spanning GDGTs content at higher temperatures at the expense of DGDs (Yosuke Koga 2012). Membranes of archaea typically show a low phase transition temperature and a broad melting behavior (Siliakus et al. 2017), which is attributed to the presence of the isoprenoid chains, their length, degree of saturation, and the positioning of methyl groups (Driessen and Albers 2007; Dannenmuller et al. 2000). In psychrophilic archaea, an increase in unsaturation of the isoprenoid chains has been observed as an adaptation to cold temperatures or increasing salinity (Dawson et al. 2012; Dong and Chen 2012). Saturated membranes provide resistance against hydrolysis and oxidation, aiding the survival of archaea in extreme environments (Sasaki et al. 2011). In bacterial membranes, the degree of saturation and variations in the length of fatty acid chains are adaptations that maintain the viscosity of membranes in response to varying temperatures, also termed homovisceous adaptation. Similar suggestions have been made for archaeal membranes (Siliakus et al. 2017), which was confirmed experimentally for one archaeal psychrophile and three halophiles. *Methanococcoides burtonii* is a psychrophilic archaeon which produces unsaturated species of archaetidylglycerol (AG), archaetidylinositol (AI), and hydroxyarchaeol (Ar_OH_) only when grow at 4 °C, but not when grown at its optimum temperature of 23 °C (Nichols et al. 2004). The genome of this psychrophile contains a single copy of the GGR and four paralogs (Allen et al. 2009); however, it lacks homologs of fatty acid desaturases from bacteria and eukaryotes, which can catalyze the addition of double bonds (Saunders et al. 2003; Goodchild et al. 2004). Later, a putative phytoene desaturase from *Methanosarcina acetivorans* was investigated; however, this enzyme was found to be responsible for the biosynthesis of hydroxyarchaeols (Mori et al. 2015).

Halophiles, such as *Natronomonas pharaonis, Haloferax sulfurifontis, and Halobaculum gomorrense*, demonstrate a strong correlation between optimal growth salinity and the fraction of unsaturated DGDs in their membranes, except for *Halohabdus utahensis* (Dawson et al. 2012). Polyextremophiles (psychrophiles and halophiles), such as *Halohasta litchfieldiae* and *Halorubrum lacusprofundi*, synthesize lower levels of GGR in response to growth at 4 and 10 °C (Williams et al. 2017). Additionally, the levels of hydroxymethylglutaryl-CoA (HMG-CoA) synthase from the mevalonate pathway were elevated in the proteomes of these organisms at cold temperatures (Williams et al. 2017). Notably, unsaturated DGDs are not exclusive to psychrophiles or halophiles and can also be found in hyperthermophilic methanogens, such as *Methanopyrus kandleri* and *Thermococcus* sp. (Sprott et al. 1997; Hafenbradl et al. 1993; Gonthier et al. 2001).

GGRs are flavoenzymes found in archaea, plants, and photosynthetic bacteria (Wang et al. 2014; Tsukatani et al. 2022; Nishimura and Eguchi 2006). They are promiscuous and can catalyze the partial or complete saturation of a variety of substrates, including DGGGP, GGPP, FPP, farnesol (FOH), and geranylgeraniol (GOH) (Sasaki et al. 2011; Meadows et al. 2018). Because of their promiscuity, they have been of interest as biocatalysts to reduce polyterpenes of various chain lengths (Kung et al. 2014; Cervinka et al. 2021). Archaeal GGRs reduce only three distal C** = **C bonds of their natural substrate GGPP, whereas GGGP derivatives can be fully saturated (Sasaki et al. 2011) (Fig. 1). Remarkably, they were able to reduce all double bonds when the substrate was elongated with a succinate spacer region (Cervinka et al. 2021). The introduction of a spacer region allows for binding to the anion binding site of the enzyme while presenting proximal double bonds to the active site, leading to the full reduction of the substrate (Cervinka et al. 2021). Thus, the chain length of the substrate appears to be a regulatory factor in the extent of saturation.

**Fig. 2:**
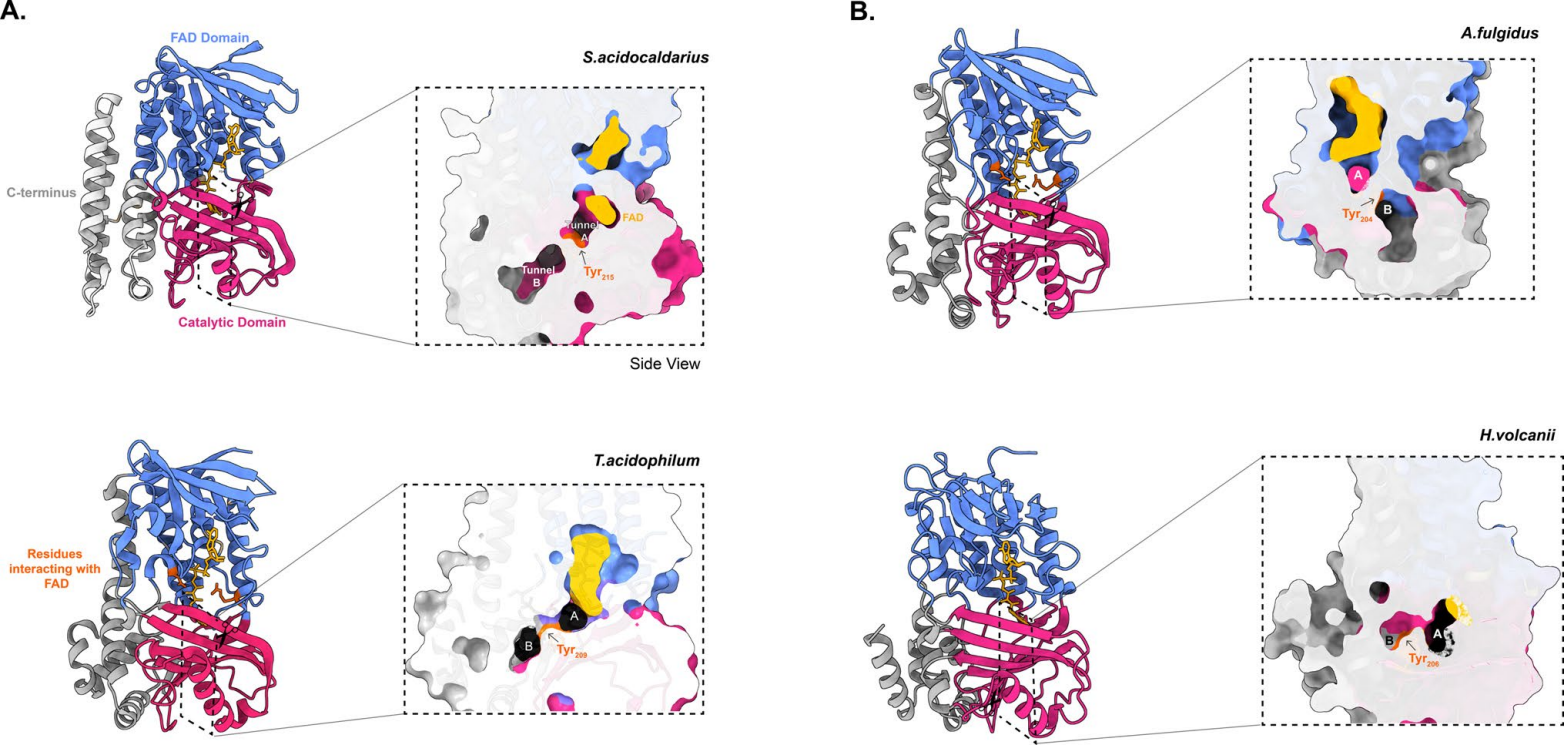
Structures of archaeal GGRs. **A** Crystal structures of *S. acidocaldarius* (PDB: 3ATQ) and *T. acidophilum* (PDB: 3OZ2). Blue, pink, and gray represent the domains. A cross-section across the surface of the catalytic cavity is shown in the boxes with dotted lines. FAD—yellow, tyrosine surface—orange, and black—ligand. **B** AlphaFold predictions of *A. fulgidus* and *H. volcanii*. These models were enriched for co-factors and ligands (FAD and lipid molecules) with AlphaFill (Hekkelman et al. 2023). All structures were visualized and annotated using ChimeraX (Pettersen et al. 2004)

GGRs from *Archaeoglobus fulgidus, Thermoplasma acidophilum*, and *Methanosarcina acetivorans* have been successfully expressed, purified, and characterized in *Escherichia coli* (Murakami et al. 2007; Ogawa et al. 2014). *M. acetivorans* GGR requires a specific in vivo reducer to catalyze the reduction of DGGGP, which is the native ferredoxin-encoding gene that is localized in the same genomic locus and displays regiospecificity for the ω-terminal double bond (Ogawa et al. 2014). The in vivo reducers of *A. fulgidus* and *S. acidocaldarius* remain unknown; thus, sodium dithionite was used in in vitro reactions (Murakami et al. 2007; Sasaki et al. 2011). *Thermoplasma acidophilum* GGR requires NADP as a reducer (Nishimura and Eguchi 2006). So far, the crystal structures of only two archaeal GGRs are available: *Sulfolobus acidocaldarius* and *T. acidophilum* (Sasaki et al. 2011; Nishimura and Eguchi 2006) (Fig. 2). Both the crystal structures were obtained in complex with FAD (Sasaki et al. 2011; Nishimura and Eguchi 2006) (Fig. 2). Some sequence motifs are conserved throughout all archaeal GGRs. Examples include GxGxxG (NAD-binding domain) and PxxxWxFP (catalytic domain) (Fig. 2) (Murakami et al. 2007). Motifs associated with FAD interaction, such as LxGD and RxxxD, are conserved in *A. fulgidus*, *T. acidophilum*, and *M. acetivorans* (Murakami et al. 2007). Additionally, the CGGG motif which interacts with the isoalloxazine ring of the FAD has been reported only in *T. acidophilum* (Fig. 2) (Murakami et al. 2007). However, the Cys47 residue itself is conserved throughout all archaeal GGRs and is essential for catalysis in *the S. acidocaldarius* enzyme (Fig. 2) (Sasaki et al. 2011). This cysteine is speculated to be involved in electron transfer or modulation of the reactivity of flavin (Xu et al. 2010). The ligand-binding domain of archaeal GGRs consists of two tunnels: A and B (Sasaki et al. 2011; Xu et al. 2010). Tunnel A is narrow and restrictive, whereas tunnel B is more permissive in its size (Sasaki et al. 2011; Xu et al. 2010) (Fig. 2). Tunnel B harbors hydrophobic amino acid residues and tunnel A is close to the isoallozaxine ring of FAD (Sasaki et al. 2011; Xu et al. 2010) (Fig. 2). Both tunnels are separated by a tyrosine residue (Sasaki et al. 2011; Xu et al. 2010) (Fig. 2). The catalytic motif PxxxWxFP is close to the FAD ring and forms a β-strand with hydrophobic residues, such as tyrosine, phenylalanine, and tryptophan (Sasaki et al. 2011; Xu et al. 2010). The positioning of these residues forms a cavity that can accommodate the geranyl group and ensures the optimal positioning of the unsaturated substrate toward FAD for reduction (Sasaki et al. 2011; Xu et al. 2010). The FAD ring undergoes conformational changes depending on its reduction state (Sasaki et al. 2011; Xu et al. 2010). The arginine residue from RxxFD motif interacts with the O4′ (3.0 Å) and O2′ (3.1 Å) of the FAD; this motif is placed opposite to the LxGD in the *T. acidophilum* structure (Xu et al. 2010) (Fig. 2). Therefore, it is proposed that these residues likely play a role in the conformational switch of FAD (Xu et al. 2010). Interestingly, the *S. acidocaldarius* GGR lacks these motifs (Sasaki et al. 2011).

Although GGRs act on lipophilic substrates, they do not contain transmembrane domains. Nevertheless, the *T*. *acidophilum* GGR was expressed and purified from the membrane fraction of *E. coli* (Nishimura and Eguchi 2006). Remarkably, only the *S. acidocaldarius* GGR contains amphipathic α-helices at its C-terminus, which are thought to mediate membrane association (Fig. 2) (Sasaki et al. 2011; Kung et al. 2014). Interestingly, this structural motif is shared by a set of lipid-synthesizing enzymes including the Tes and the cyclopropane fatty acid synthase from *E. coli* (Lloyd et al. 2022). This structure seems to act as lipid pocket that is lined solely by hydrophobic residues (Lloyd et al. 2022). The *S. acidocaldarius* structure also contains a disulfide bridge formed by Cys310 and Cys335, which is unique to GGR (Sasaki et al. 2011).

## Multiplicity of GGRs in archaeal genomes

**Fig. 3:**
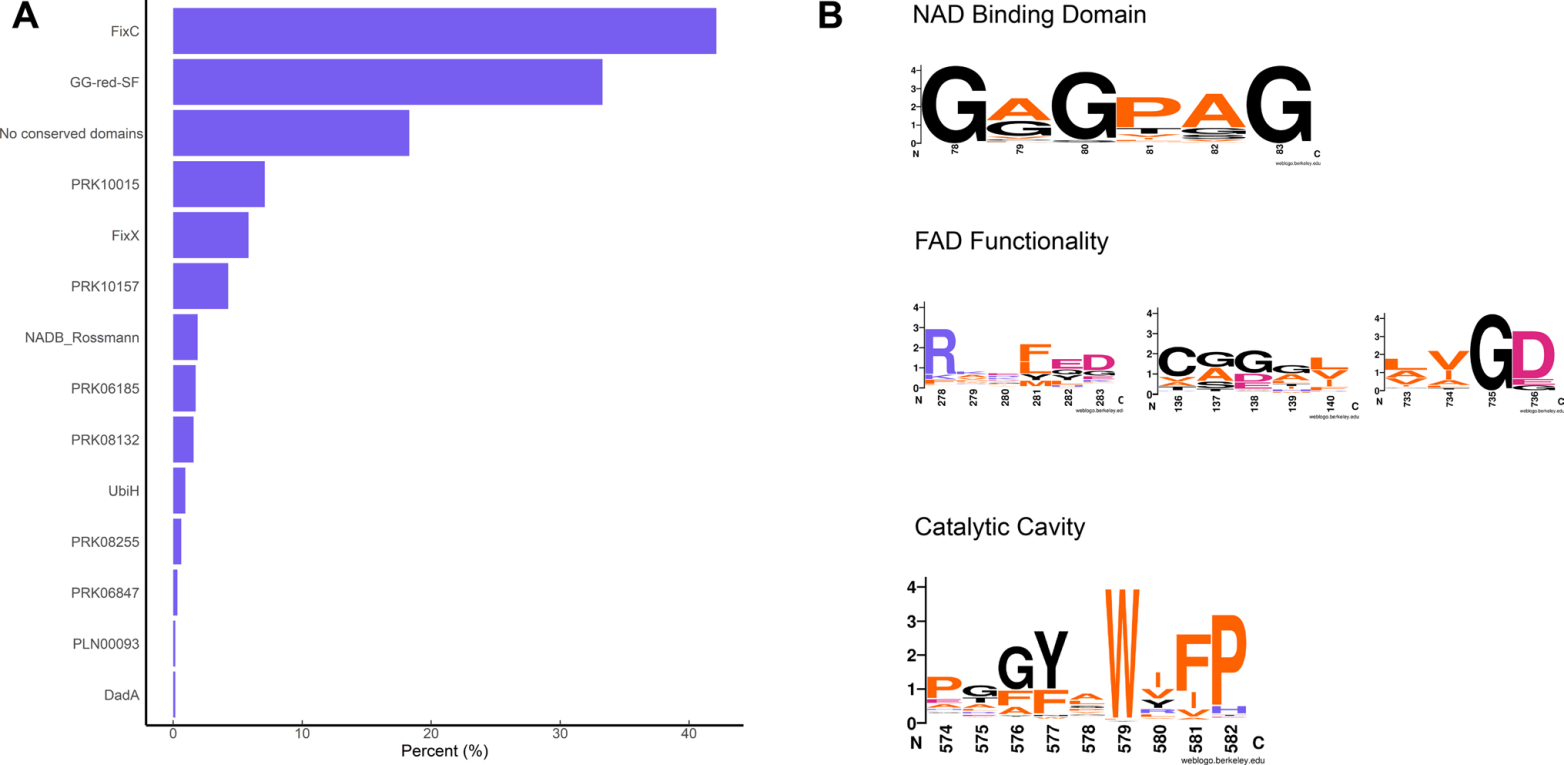
Overview of **A** conserved domains and **B** sequence motifs associated with GGRs and paralogs in arCOG00570 (*n* = 750). Sequence motif logos were generated using WebLogo (Crooks et al. 2004)

Apart from membrane phospholipids, archaea also harbor isoprenoid-based polyterpenes, such as carotenoids, apolar polysioprenoids, polyprenyl phosphates, and quinones, whose complete biosynthetic pathways are not yet known (Salvador-Castell et al. 2019). These polyterpenes are found in various saturation states in archaeal membranes, and some have been proposed to modulate membrane properties (Salvador-Castell et al. 2019). Most archaea have a multiplicity of GGRs in their genomes, which are clustered in the archaeal orthologous cluster of genes arCOG00570 (Hernández-Plaza et al. 2022; Makarova et al. 2015). Currently, this cluster consists of 2024 genes from 452 archaeal species (Makarova et al. 2015). Investigation of some of these proteins from *A. fulgidus* led to the discovery of menaquinone (MK)-specific prenyl reductase (AF_PR) (Hemmi et al. 2005). This enzyme was found to be responsible for producing partially saturated side chains of octaprenyl MK in *E. coli* (Hemmi et al. 2005). Interestingly, this enzyme does not have any predicted transmembrane domains nor does it contain the catalytic and FAD-associated GGR motifs (Hemmi et al. 2005) (Fig. 3). Other paralogs from *A. fulgidus* were also investigated in this study; however, their expression did not lead to any alteration in the quinone profile of *E. coli* (Hemmi et al. 2005). Such GGR paralogs have also been found in mycobacteria, one of which was recently identified as heptaprenyl reductase (HepR) (Abe et al. 2022). HepR was able to reduce ω- and *E*- prenyl units in Z,E-mixed heptaprenyl diphosphates from *Mycolicibacterium vanbaalenii* (Abe et al. 2022). The majority of GGR paralogs from archaea remain uncharacterized, and their presence has been correlated with the saturation states of polyterpenes in archaeal membranes (Guan et al. 2011).

The focus of this review is on the uncharacterized GGR paralogs present in the genomes of extremophilic archaea. These will be discussed through structural information obtained through AlphaFold2 modeling, analyses of the genomic loci, conserved domains, extant protein, or transcript expression datasets from these organisms, and where possible correlated to specific terpenoids or isoprenoids.

## Conserved sequence and structural motifs in archaeal GGR paralogs

A batch conserved domain analysis indicated that 40% of these proteins had a FixC domain (flavoproteins, such as dehydrogenase), 33% had a GG-red-SF domain (containing the GGR family), 18.3% did not contain any conserved domains, and only 5% had the NADB_Rossman domain (Fig. 3a). The latter is found in the FAD domain of all identified archaeal GGRs (Xu et al. 2010). The GxGxxG motif that is associated with NAD binding showed high sequence conservation in this arCOG (Fig. 3b) (Lesk 1995). The entire PxxYxWxFP motif associated with the catalytic cavity of GGRs is not highly conserved in this arCOG (Fig. 3b). However, the hydrophobic residues tyrosine, tryptophan, and phenylalanine were quite well conserved (Fig. 3b). As previously described, these residues play a critical role in determining the shape of the catalytic cavity in archaeal GGRs. Low sequence conservation was observed for the LxGD and RxxFD motifs, which are involved in FAD interaction (Fig. 3b). The arginine from the RxxFD motif is highly conserved in this cluster and is likely associated with the interaction and conformational switch of FAD (Fig. 3b).

**Fig. 4:**
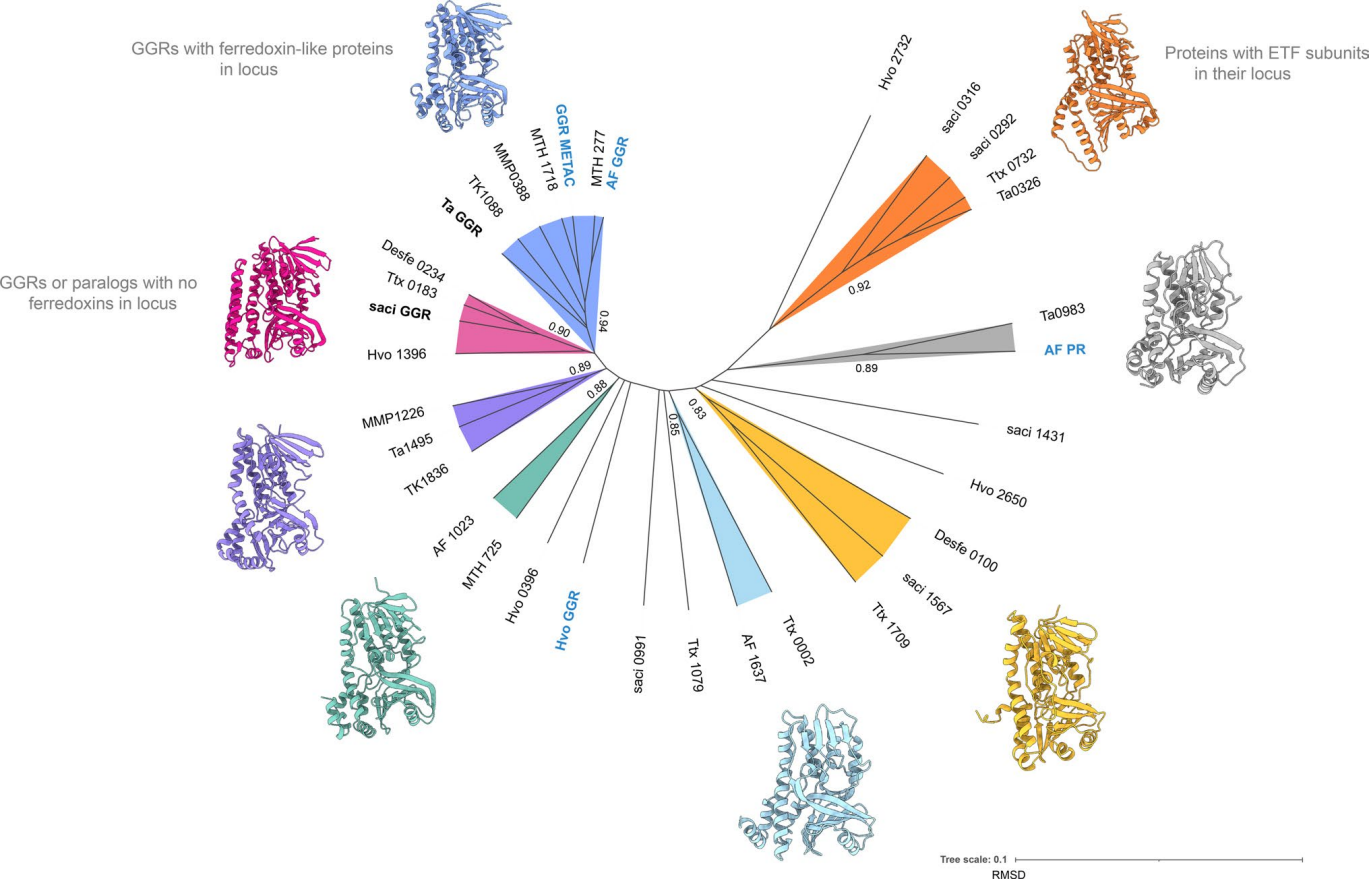
Root-mean-square deviation (RMSD) tree of archaeal GGRs and their paralogs. Proteins with available crystal structures are indicated in bold. Functionally characterized proteins (through heterologous expression or gene deletion) are denoted in blue and bold, respectively. The remaining structures were generated using AlphaFold2 (Jumper et al. 2021). mTM-Align was used as an algorithm for structural imposition and the tree was constructed using the neighbor joining method in PHYLIP (Dong et al. 2018; Eguchi 2011). TM scores for structural impositions are indicated at the nodes. Tree was visualized, annotated through iTOL and UCSF Chimera (Letunic and Bork 2021; Pettersen et al. 2004)

**Fig. 5:**
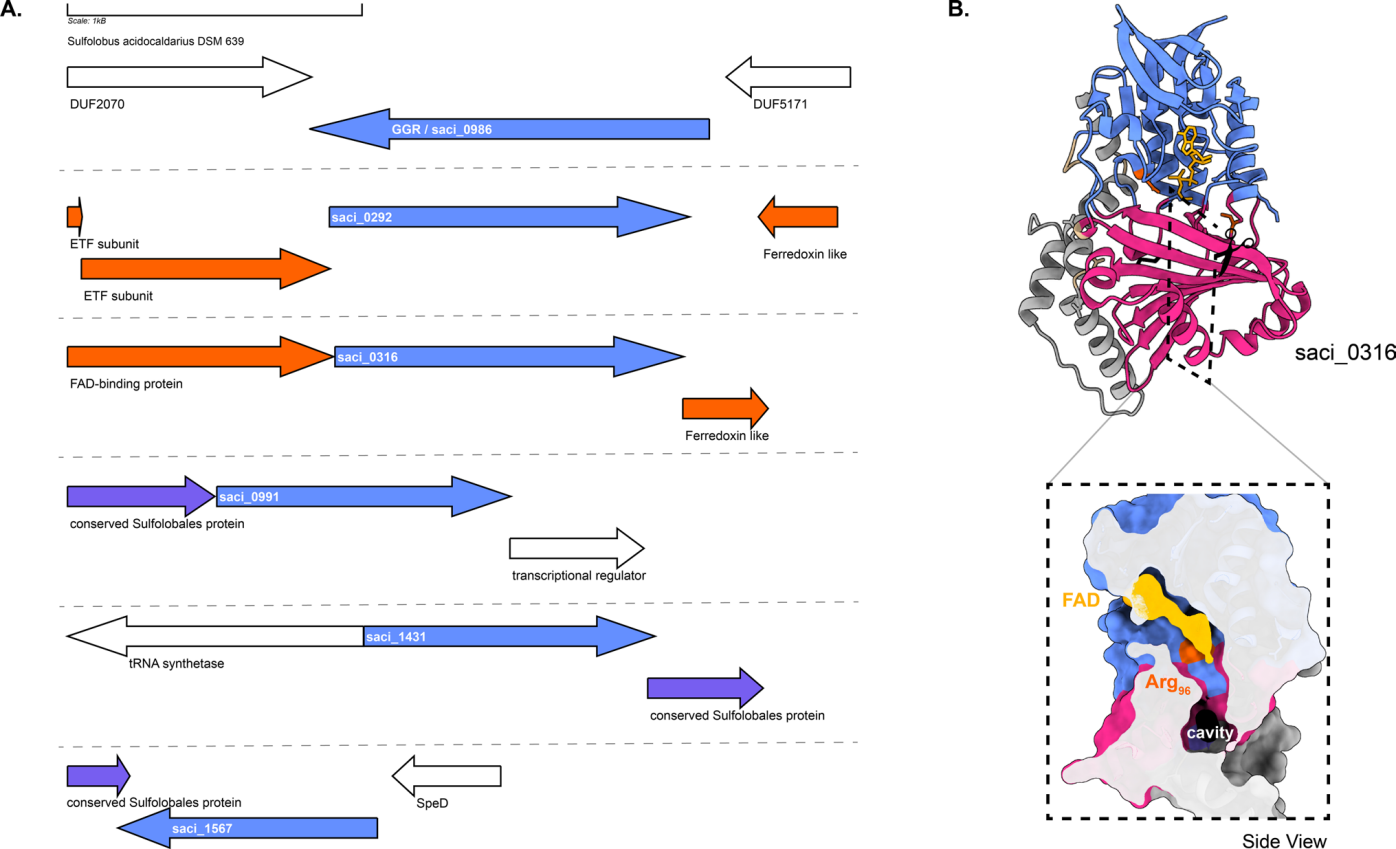
Genomic locus of *S. acidocaldarius* GGR and its paralogs, modeled structure of saci_0316 with a cross-section across its surface: **A** Figure was generated using GeneGraphics (Harrison et al. 2018). *ETF* electron transfer flavoprotein, *SpeD* S-adenosylmethionine decarboxylase. FAD binding/ETF/ferredoxin-like proteins are colored orange. Conserved proteins in the same order are colored purple. Annotations have been added based on conservation information from the arCOGs or Uniprot. **B** Colors in the Alphafold2 model structure indicate domains, and the color scheme is the same as that in Fig. 2

To understand the structural conservation among these paralogs, a root-mean-square deviation (RMSD) tree was constructed from extremophilic archaeal model organisms using mTM-align (Fig. 4) (Dong et al. 2018). For this RMSD tree, the available crystal structures of archaeal GGRs and structure models from AlphaFold were used as the input (Jumper et al. 2021). Structural alignment shows diversity among these paralogs. Notably, GGR or paralogs with ferredoxin-like proteins in their genomic loci tend to cluster together (Fig. 4, orange and blue). Similarly, GGRs or paralogs with no such proteins in their loci clustered among themselves (Fig. 4, pink). In the following sections, these paralogs are discussed in more depth based on their structural models, conservation of motifs, and genomic loci from extremophilic archaeal species. The predicted structures of the representative proteins from the various colored nodes (Fig. 4) were supplemented with co-factors and/or ligands with AlphaFill (Figs. 5b,7b,8b,9b,10b,11b,14b) (Hekkelman et al. 2023).

## GGR paralogs from Sulfolobales

The order Sulfolobales consists of sulfur-metabolizing, strictly aerobic thermoacidophiles in crenarchaeota (Liu et al. 2021). The membrane of Sulfolobales is composed of ~ 99% monolayer forming GDGTs and GDNTs (Jensen et al. 2015). GDGTs are the dominant species in the membrane, whereas GDNTs are less abundant (Jensen et al. 2015). Deletion of the calditol synthase (Cds) in *S. acidocaldarius*, and hence the loss of GDNTs in the membrane, rendered cells lethal to a low pH (1.0) environment; thus, GDNTs have been proposed to aid in acidic stress (Zeng et al. 2018). Bilayer-forming DGDs constitute a minor fraction of the membrane (Jensen et al. 2015). The common polar headgroups found in membrane phospholipids include: monohexose, di-hexose, penta-hexose, and sulfoquinovose (Jensen et al. 2015; de Kok et al. 2021). Interestingly, the presence of carotenoids has only been reported in a natural mutant of *Sulfolobus shibatae* (Kull and Pfander 1997). The carotenoid species were identified as C_50_ (*Z*)-isomers of zeaxanthin and were proposed as membrane reinforcers (Kull and Pfander 1997).

*S. acidocaldarius* (optimum: 75 °C, pH 3.0) is a model organism from the order of Sulfolobales and its membrane has been studied extensively. The most common adaptations of the membrane include the incorporation of cyclopentane rings (0–8) and altering the ratio of GDGTS to DGDs (Rastädter et al. 2020b; Quehenberger et al. 2020). The latter is dependent on the growth phase and rate (Quehenberger et al. 2020). Specifically, an increase in the growth rate is correlated with an increased ratio (from 1:3 to 1:5) of GDGTs to DGDs and a decrease in the average number of cyclopentane rings (from 5.1 to 4.6) (Quehenberger et al. 2020). The incorporation of pentacyclic rings is also affected by the availability of nutrients and growth phase (Quehenberger et al. 2020; Bischof et al. 2019). Under nutrient-rich conditions, the overall number of cyclopentane rings decreases, leading to increased permeability, whereas under conditions of nutrient depletion, the overall number of rings remains unchanged (Bischof et al. 2019). The organism also produces species of respiratory quinones, such as caldariellaquinone (CQ) and sulfoquinone (SQ), membrane anchors, such as dolichol phosphate (DoIP), and C_20_-C_35_ apolar polyisoprenoids (Salvador-Castell et al. 2019; Holzer et al. 1979; Elling et al. 2016). Typically, CQ and SQs are found in their fully saturated forms, and their production is dependent on the amount of oxygen (Elling et al. 2016; Trincone et al. 1989). Fully saturated species of α- and ω-of C_40_,C_45_, and C_50_ DoIP species have been found in *Sulfolobus* (Guan et al. 2011). Partially saturated species of C_45_ DoIP with three to eight double bonds have also been reported (Guan et al. 2016). However, the saturation profiles of C_20_-C_35_ polyisoprenoids remain elusive (Holzer et al. 1979).

The crystal structure of GGR from *S. acidocaldarius* consists of three distinct domains: FAD binding, catalytic, and a C-terminal domain (Sasaki et al. 2011) (Fig. 2). This GGR does not have a ferredoxin-encoding gene near its genomic locus (Fig. 5a). There are five uncharacterized paralogs of GGRs in this species, saci_0292 (PRK10015 superfamily, provisional oxidoreductases), saci_0316 (FixC superfamily), saci_0991, saci_1431, and saci_1567. All these paralogs are well expressed in the stationary growth phase of *S. acidocaldarius* (Cohen et al. 2016). Notably, none of these paralogs clustered with *the S. acidocaldarius* GGR in the RMSD tree (Fig. 4).

**Fig. 6:**
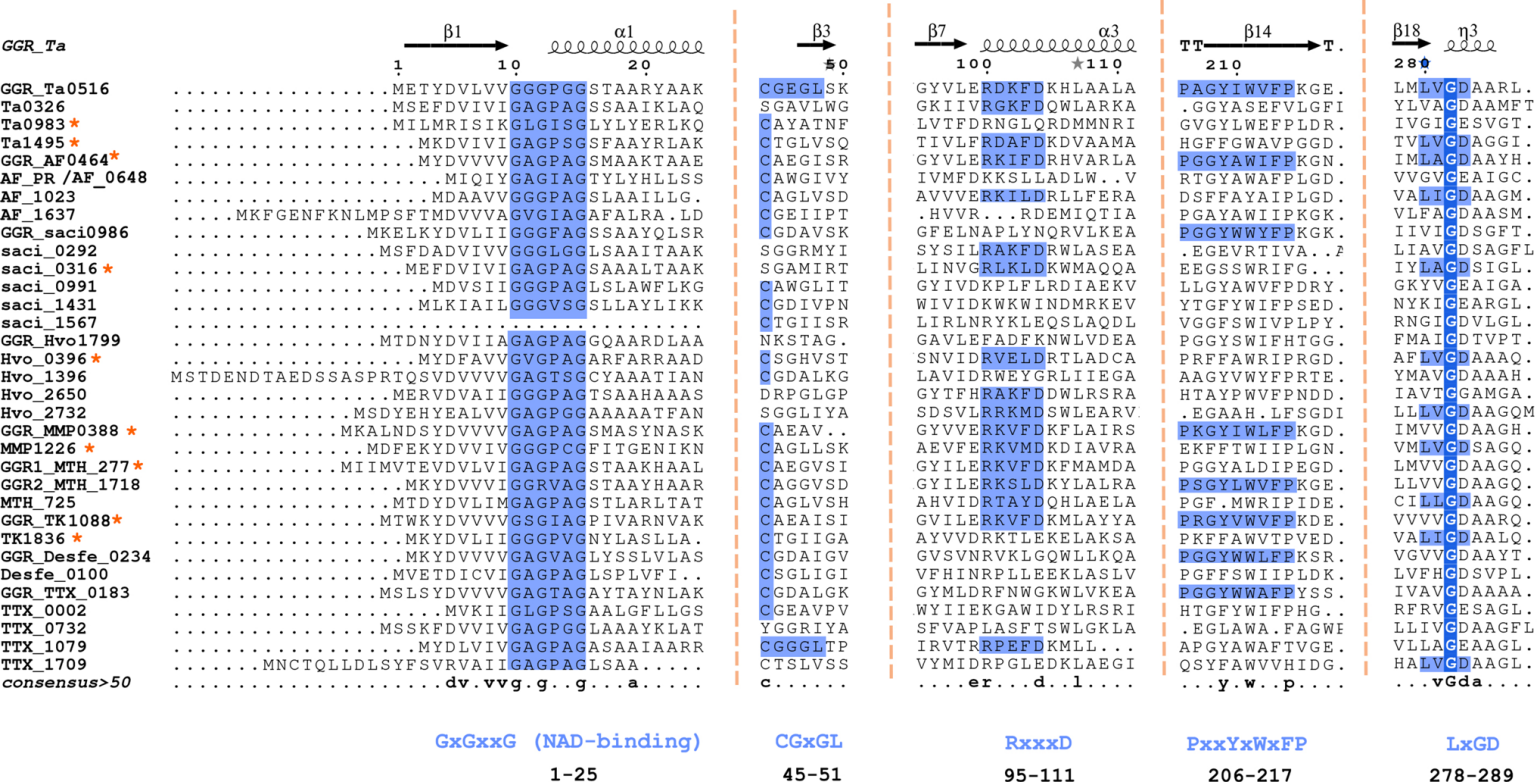
Multiple sequence alignment of representative extremophilic archaeal GGRs and paralogs. Multiple sequence alignment was performed using Clustal Omega and annotated on the ENDscript server (Robert and Gouet 2014). Orange asterisks indicate proteins with one predicted transmembrane segment in their structures (computed using DeepTMHMM) (Hallgren et al. 2022)

Saci_0316 is in the vicinity of genes encoding an FAD-binding protein and a ferredoxin family protein (Fig. 6). Saci_0292 has two predicted electron transport flavoprotein (ETF) subunits in its cluster: saci_0290 (arCOG00446) and saci_0291 (arCOG00447) (Fig. 6). Meanwhile, saci_0293 is a predicted ferredoxin-like protein conserved only in crenarchaeota (arCOG01985) (Fig. 6). Saci_0292 and saci_0316 share the node with other paralogs in the RMSD tree, which also harbors ferredoxins or *other* subunits in their genomic locus (Fig. 4). These proteins contain the RxxxD motif, whereas saci_0316 contains the LxGD motif (Fig. 6). A cross-section across the surface of the model structure of saci_0316 shows the presence of a cavity (Fig. 5b). Interestingly, the Cys47 residue (essential for catalysis in *S. acidocaldarius* GGR) was conserved only in saci_0991, saci_1431, and saci_1567 (Fig. 6). Meanwhile, Saci_0991 and Saci _1567 have conserved Sulfolobales proteins in their genomic loci (Fig. 6). Saci_0316, saci_1431, and saci_1567 are suggested to encode for essential proteins based on a genome wide transposon mutagenesis study in *S. islandicus* (Zhang et al. 2018).

## Archaeoglobales

Archaeoglobales are sulfur-metabolizing hyperthermophiles that are strict anaerobes (Beeder et al. 1994). Organisms belonging to this order can be found in hydrothermal environments, such as vents, oil wells, and springs (Beeder et al. 1994). *Archaeoglobus fulgidus* is a model organism of this order and is slightly halophilic (1.9% NaCl, wt/vol) (Borges et al. 2006). The membrane of *A. fulgidus* is primarily composed of archaeols, caldarchaeols, and MK-7 (Koga and Morii 2005; Tarui et al. 2007; Lai et al. 2008). Archaeol species include AA, archaetidylinositol (AI), and diglycosylarchaeol (DGA), and caldarchaeol species include caldarchaetidylinositol (CI), caldarchaetidic acid (CA), diglycosylcaldarchaeol (DGC), and diglycosylcaldarchaetidylinositol (DGCI) (Tarui et al. 2007). Additionally, an unidentified polar lipid is present in the membrane (Tarui et al. 2007). C_55_, C_60_, and C_65_ heptasaccharide-charged dolichol phosphates have been reported in *A. fulgidus* membranes (Taguchi et al. 2016). The organism responds to heat and osmotic stress by increasing the content of di-myo-inositol phosphate (D1P) and diglycerol phosphate (DGP), which are rare osmolytes found only in Archaeoglobales and *Aquifex* bacteria (Borges et al. 2006; GonÃ§alves et al. 2003).

**Fig. 7:**
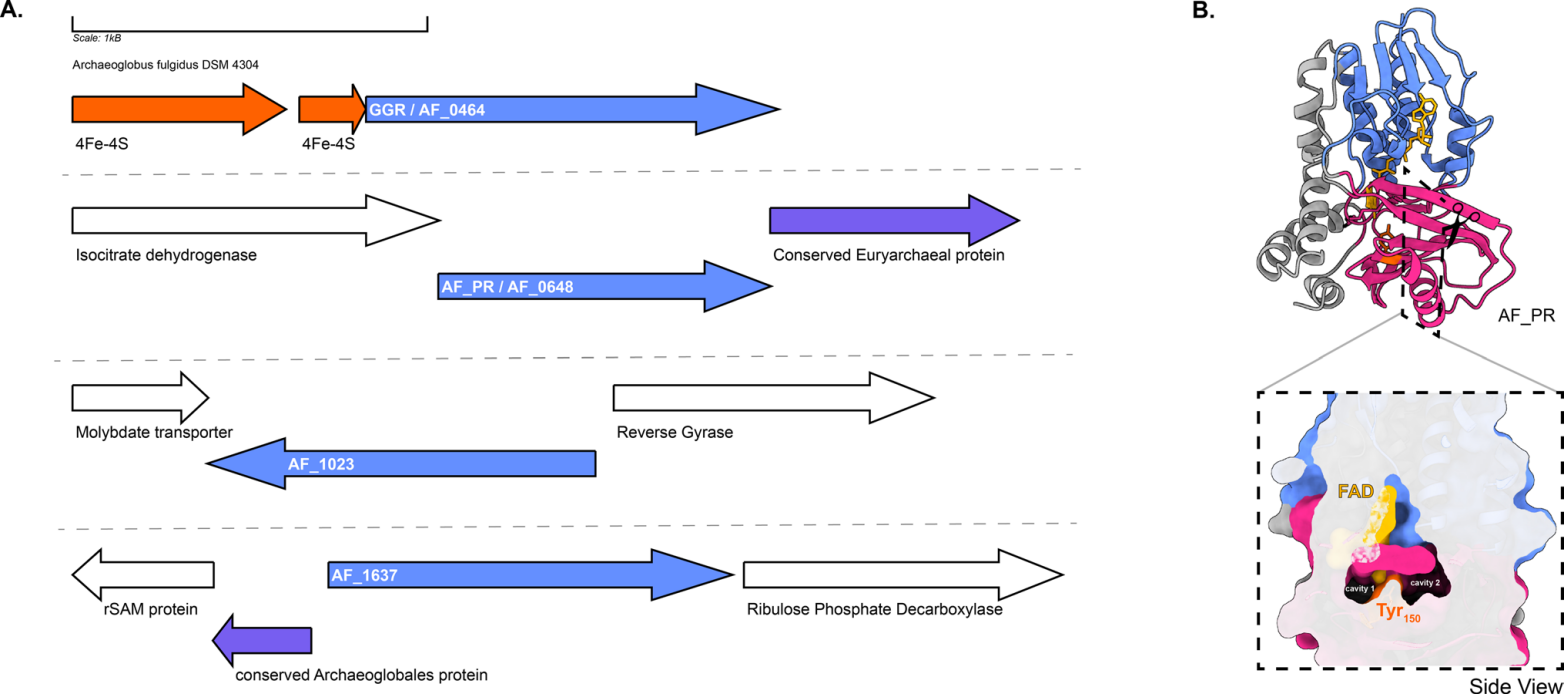
The genomic locus of *A. fulgidus* GGR and its paralogs, modeled structure of AF_PR: **A** Figure was generated using GeneGraphics (Harrison et al. 2018). FAD binding/ETF/ferredoxin-like proteins are colored orange. Conserved proteins in the same order or family are colored purple. Annotations have been added based on conservation information from the arCOGs or Uniprot. **B** AlphaFold2 model of AF_PR along with cross-section across its surface. The color scheme is the same as that shown in Fig. 2

GGR from *A. fulgidus* has been characterized by heterologous expression in *E. coli* (Murakami et al. 2007). The enzyme noncovalently binds to FAD (Murakami et al. 2007). Sodium dithionite and not NADPH is required as a reducing agent in the in vitro reaction for the reduction of DGGGP to AA (Murakami et al. 2007). This indicates that the enzyme likely accepts electrons from specific reducing agents, such as ferredoxins or cofactor F_420_ (Murakami et al. 2007). The locus of the gene encoding GGR contains a predicted 4Fe-4S protein upstream, AF_RS02355 (WP_048064240.1), which could possibly function as a reducer (Fig. 7a). The *A. fulgidus* GGR clustered with the *Thermoplasma acidophilum* GGR in the RMSD tree, which also contained a 4Fe-4FS encoding gene downstream in the locus (Figs. 4 and 7b). The structural model of this GGR has a similar organization of the catalytic cavity as other archaeal GGRs (Fig. 2). This organism contains three paralogs of GGR: AF_0648 (PR), AF_1023 (FixC superfamily), and AF_1637 (GG-red-SF superfamily). One archaeal GGR paralog (AF_0648) was characterized by *A. fulgidus* (Hemmi et al. 2005). The paralog was found to be responsible for the production of partially saturated derivatives of menaquinone-8 (MK-8) when expressed in *E. coli* and annotated as a menaquinone-specific PR (Hemmi et al. 2005). A cross-section across the surface of the modeled AF_PR indicates two cavities separated by Tyr150 (Fig. 7b). Cavity 1 was in close proximity to the introduced FAD cofactor in this model (Fig. 7b). This structural organization is similar to that of the archaeal GGRs (Fig. 2). Other paralogs (AF_1023 and AF_1637) were also expressed in *E. coli*, but this did not lead to any alteration in the quinone profiles (Hemmi et al. 2005). AF_1637 is an interesting uncharacterized protein, as it contains the PxxYxWxFP catalytic cavity associated with GGRs along with a conserved Archaeoglobales protein upstream in its locus (Figs. 6 and 7b). AF_1023 contained RxGD and LxxxD motifs associated with FAD interaction (Fig. 6).

## Haloferacles

The order Haloferacles comprises halophiles that thrive in environments approaching salt saturation, such as natural brine, alkaline salt lakes, hypersaline lakes, and marine solar salterns (Stan-Lotter and Fendrihan 2015). The membranes of these organisms contain bilayer-forming glycolipids, phospholipids, cardiolipins, carotenoids such as bacterioruberin (monolayer-like), and respiratory quinones such as menaquinone (Kellermann et al. 2016). *Haloferax volcanii* (optimum: 45 °C, 2 M NaCl) is a model organism from this order. This halophile is pleomorphic and it has been suggested that it utilizes various shapes to create turbulence in the membrane, leading to efficient diffusion (Kellermann et al. 2016). The membranes of halophiles harbor the highest content of respiratory quinones known in archaea (Kellermann et al. 2016). In particular, their membranes are dominated by MK (relative abundance:65%) and bilayer-forming glycerolipids such as AG (Kellermann et al. 2016). The impact of such a high MK content on the physiochemical properties of the haloarchaeal membrane is not known. However, molecular dynamics simulations have suggested that MK-8 increases membrane thickness, thereby increasing the membrane bending constant (Feng et al. 2021). This, in turn, allows the membrane to resist shrinkage in a hypersaline environment (Feng et al. 2021).

These membranes are one of the most negatively charged among all domains of life, considering that they also contain cardiolipins (CLs) and methylated archetidylglycerophosphate (AGP-Me) (Bale et al. 2019; Stan-Lotter and Fendrihan 2015). The divalent negative charge of polar head groups such as AGP-Me stabilizes the trimeric structure of bacteriorhodopsins (bRs) found in halophilic membranes, while the branched methyl chains significantly enhance the affinity for bRs (Umegawa et al. 2023). Recently, acetylated phospholipid species such as acetylated archaeol and acetylated AG have been reported in the membranes of *H. volcanii* and *Halobacterium salinarum* (Kropp et al. 2022). They were found in various saturation states ranging from 18 double bonds in the structure (Kropp et al. 2022). The functional significance of such polar headgroup modifications in membranes remains unclear.

The elevated contents of MKs and extremely negatively charged membranes have been hypothesized to fulfill high rates of electron transport, which would be required for energy maintenance in chronic energy stresses such as high salinity (Kellermann et al. 2016; Valentine 2007). *De-novo* synthesis of CLs in *Halobacterium salinarum* has been experimentally correlated with hypotonic osmotic shock (from 4 to 0.1 M NaCl) (Lobasso et al. 2003; Lopalco et al. 2004). Glycocardiolipins are also known to interact with bacteriorhodopsin in halophilic membranes (Corcelli et al. 2004). Thus, it has been hypothesized that cardiolipins form an efficient barrier in the halophilic membrane against the high ionic levels while also stabilizing bacteriorhodopsin (Lopalco et al. 2004; Corcelli et al. 2004). Currently, the only identified and characterized archaeal cardiolipin synthase (Cls) is from *Methanospirillium hungatei*. The distribution of Cls is not exclusive to halophiles; it is also found in crenarchaeotes such as *Nitrosphaera gargensis* (moderate thermophile, optimum: 46 °C), hyperthermophiles such as *Pyrobaculum ferrireducens* (optimum: 95 °C) and *A. fulgidus* (Exterkate et al. 2021; Slobodkina et al. 2015; Pitcher et al. 2010). In *Haloferax mediterranei*, the levels of carotenoids such as bacteroruberin increase in the membrane in response to oxidative stress (Giani and Martínez-Espinosa 2020).

Membrane modulation in halophiles is through a *LonB* protease which acts on PSY, a rate-limiting enzyme in carotenoid synthesis (Cerletti et al. 2018). The contents of bacterioruberins (BRs) and a few uncharacterized polar lipids have been found to increase in *LonB* mutants (Cerletti et al. 2014). It is worth noting that a deletion mutant of GGR has only been obtained in *H.volcanii* among all archaea (Naparstek et al. 2012)*.* In the absence of GGR, the organism was unable to produce saturated dolichol phosphates and diether phospholipids (Naparstek et al. 2012). Remarkably, in this mutant, partial saturation at the α-position of the C_60_ dolichol phosphate (DoIP) occurred (Naparstek et al. 2012). Unsaturated molecules of AGP-Me and archaetidylglycerol (AG) were also detected in the lipidome of the deletion mutant (Naparstek et al. 2012). Growth and lipidomic analysis of this deletion mutant grown at various ranges of salinity and temperature could provide more insights about the role of saturated membranes in halophiles. A crystal structure of this GGR is not available; however, the enriched structural model with ligands shows a similar organization of the catalytic cavity as other archaeal GGRs (Fig. 2).

**Fig. 8:**
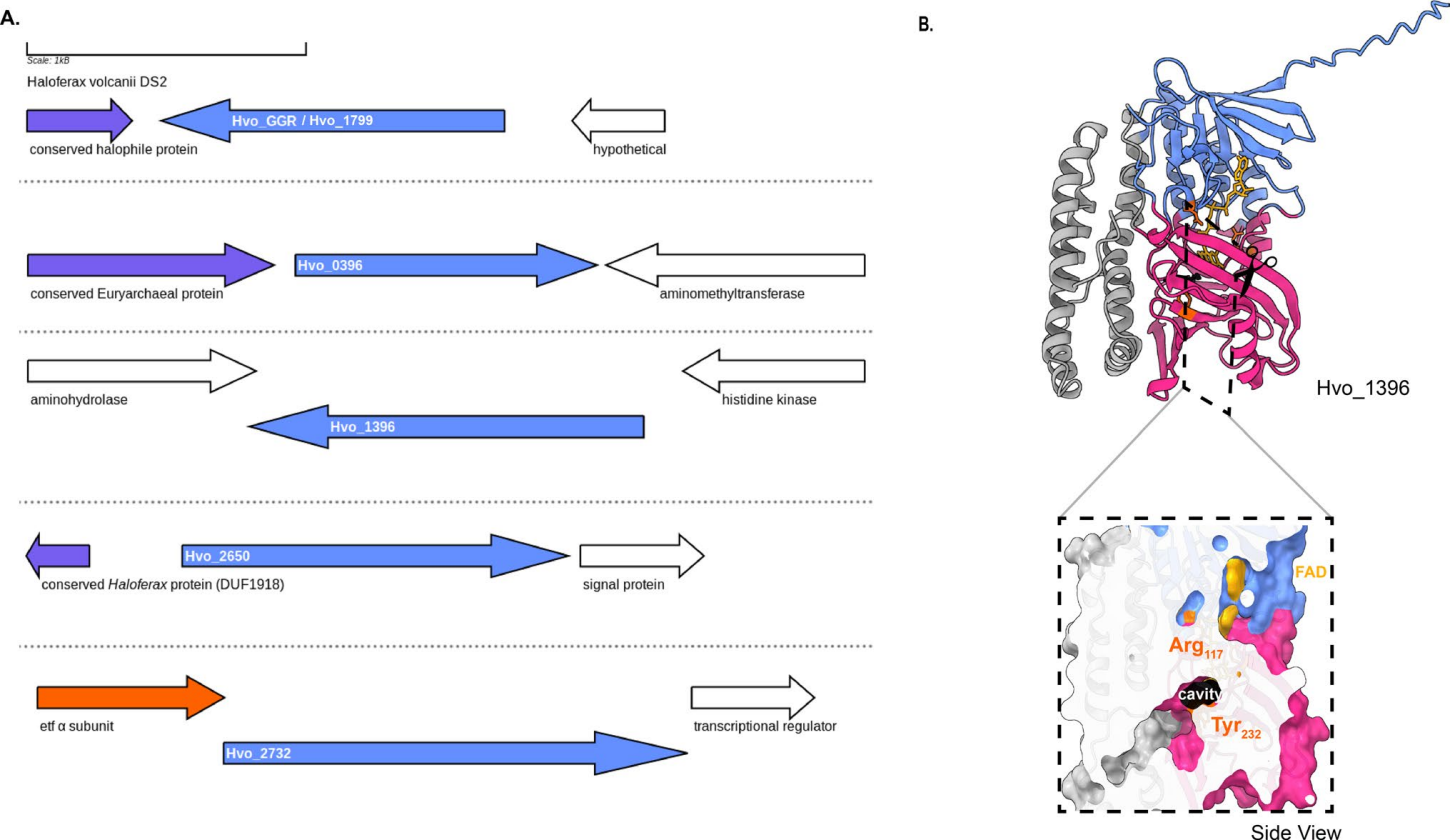
Genomic locus of *H.volcanii* GGR and its paralogs, modeled structure of Hvo_1396. **A** Figure was generated using GeneGraphics (Harrison et al. 2018). *ETF* electron transfer flavoprotein. FAD binding/ETF/ferredoxin-like proteins are colored orange. Conserved proteins in the same order are colored purple. Annotations have been added based on conservation information from the arCOGs or Uniprot. **B** AlphaFold2 enriched model and cross-section across the surface of the catalytic cavity. The color scheme is the same as Fig. 2

The genome of *H. volcanii* harbors four GGR paralogs: Hvo_0396, Hvo_1396, Hvo_2650, and Hvo_2732 and all of them are expressed under standard culturing conditions (Schulze et al. 2020). The GGR from *H. volcanii* and Hvo_2650 genes co-localize in their locus with genes encoding conserved haloarchaeal proteins (Fig. 8a). One of the paralogs—Hvo_2732—has a conserved alpha subunit of ETF encoding gene in its locus (arCOG00447) (Fig. 8a). This protein contains the following motifs: RRKMD and LVDG, both of which are associated with FAD interaction (Fig. 6) (Nishimura and Eguchi 2006). Hvo_0396 contains only the RxxxD motif (Fig. 6). All the structure predictions of GGR paralogs from this order are distinct, except Hvo_1396 which shares some structural conservation with the *S. acidocaldarius* GGR (Fig. 4). The enriched model of Hvo_1396 shows a Tyr_232_ residue separating a potential catalytic cavity (Fig. 8b).

## Thermoproteales

Thermoproteales represent a group of strict anaerobes and sulfur dependent hyperthermophiles of the crenarchaeota order (Siebers et al. 2011). *Aeropyrum pernix* (optimum: 90–95 °C, 3.5% salinity) from this group produces C_25,25_-achaetidylinositol and C_25,25_-achaetidyl(glucosyl)inositol, also known as extended archaeol (Kejžar et al. 2022). These extended archaeols are reduced by geranylfarsenylreductase (GFR) (Yoshida et al. 2018). Remarkably, *S. acidocaldarius* GGR was not able to reduce the C_25_ extended archaeols in *E. coli*, suggesting that GFR is specialized for the reduction of geranylfarsenyl groups (Yoshida et al. 2018).

**Fig. 9:**
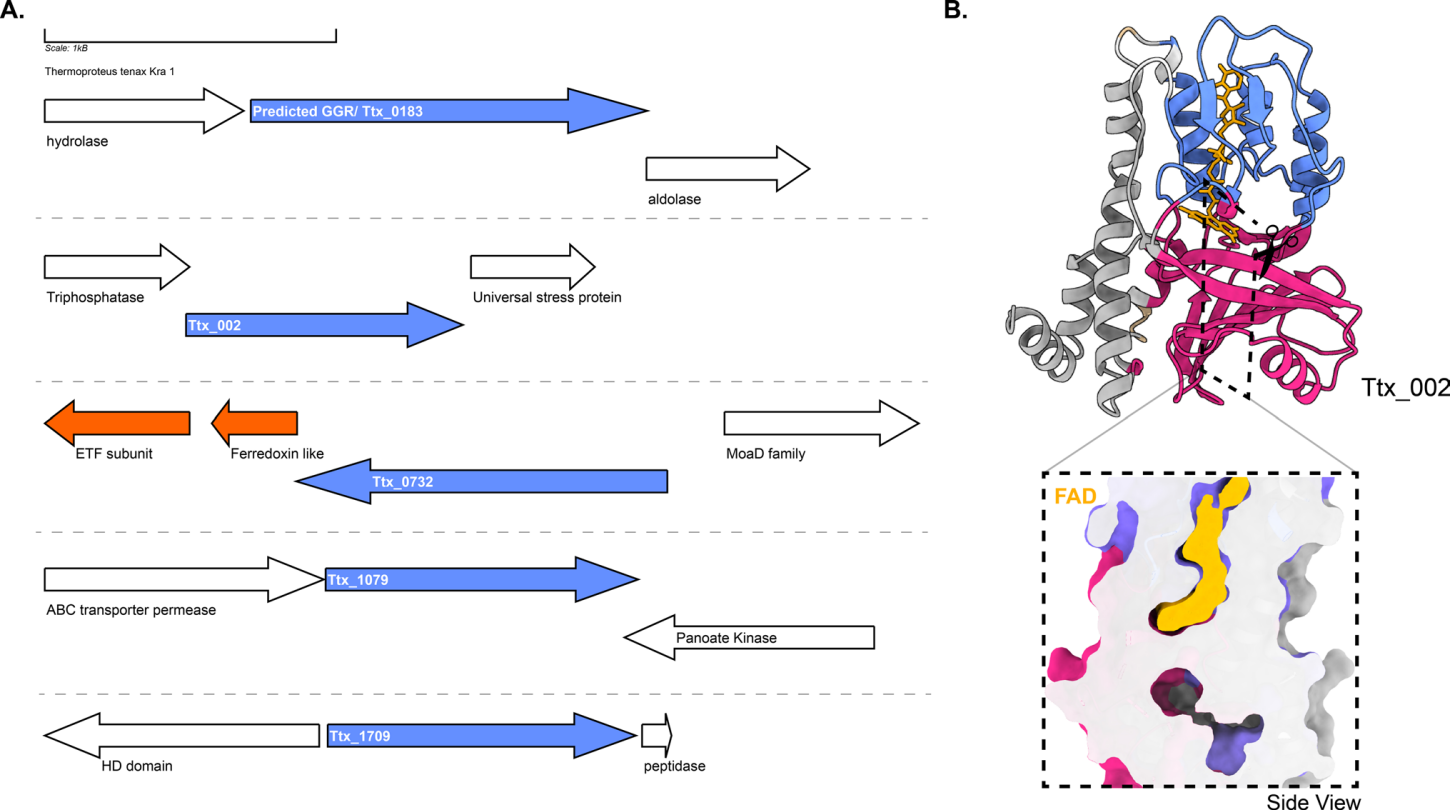
Genomic locus of *T.tenax* GGR and its paralog, structural model of Ttx_002. **A** Figure was generated using GeneGraphics (Harrison et al. 2018). *ETF* electron transfer flavoprotein, *MoaD* molybdopterin synthase. FAD binding/ETF/ferredoxin-like proteins are colored orange. Conserved proteins in the same order are colored purple. Annotations have been added based on conservation information from the arCOGs or Uniprot. **B** AlphaFold2 model and cross-section across the surface of catalytic cavity; the color scheme is the same as that shown in Fig. 2

*Thermoproteus tenax* is a model organism from this order which grows optimally at 86 °C, pH 5.6 (Siebers et al. 2011). The organism has a facultative chemolithoautotrophic metabolism (Siebers et al. 2011; Zaparty et al. 2008). Not much is known about the membrane composition of *T. tenax*; however, homologs of the membrane lipid biosynthetic pathway are found in the genome (Siebers et al. 2011). *T. tenax* utilizes MK as electron carriers in the respiratory chain (Thurl et al. 1985). Specifically, three species of MKs are found in their fully saturated and mono-saturated forms in the organism: MK-4, MK-5, and MK-6 (Thurl et al. 1985). Methylated species of MK-5 and MK-6 have also been reported in the quinone fraction (Thurl et al. 1985). *T. tenax* harbors 3 GGR paralogs: Ttx_002, Ttx_0732 (PRK10157 superfamily, putative oxidoreductases), and Ttx_1709. The predicted GGR from *T. tenax* does not have any ferredoxin-encoding gene in its locus and shares structural similarities with the *S. acidocaldarius* GGR (Figs. 4 and 6). Ttx_0732 contains a ferredoxin-encoding gene (arCOG01984, conserved in haloarchaea and a few archaea from TACK superphylum) upstream in its locus (Fig. 9a). Interestingly, the AlphaFold2 prediction of this protein clusters with other archaeal GGR paralogs which also have a ferredoxin-encoding gene in their locus (Fig. 4). The protein sequence of Ttx_1079 contains CxxxG and RxFD motifs (Fig. 6). Meanwhile, the sequence of Ttx_1709 includes the LxGD motif and is conserved structurally with Saci_1567, Desfe_0100 (Figs. 4 and 6). Remarkably, Ttx_002 shares structural similarities with AF_1637 and Ttx_1079 (Fig. 4). A cross-section across its enriched structural model shows a cavity; however, it is not in the vicinity of FAD (Fig. 9b).

## Desulfurococcales

**Fig. 10:**
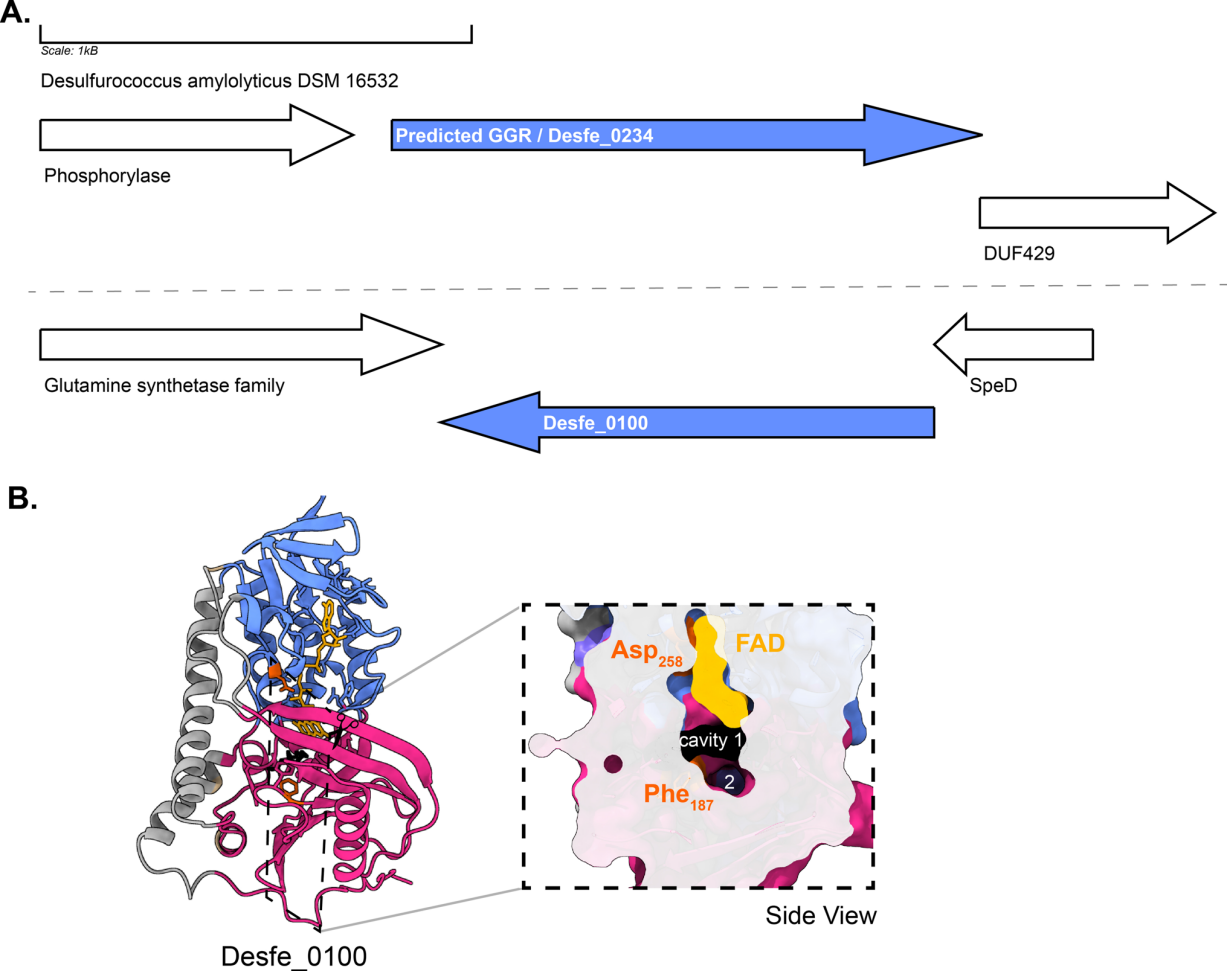
Genomic locus of *D. amylolyticus* GGR and its paralogs, modeled structure of Desfe_0100. **A** Figure was generated using GeneGraphics (Harrison et al. 2018). *ETF* electron transfer flavoprotein, *MoaD* molybdopterin synthase. FAD binding/ETF/ferredoxin-like proteins are colored in orange. Conserved proteins in the same order are colored in purple. Annotations have been added based on conservation information from the arCOGs or Uniprot. **B** AlphaFold2 enriched structure and cross-section across the protein surface. Color scheme is same as in Fig. 2

Organisms belonging to Desulfurococcales are hyperthermophiles which are obligate anaerobes and organoheterotrophs (Perevalova et al. 2005). *Desulfurococcus amylolyticus* (formerly known as *D. fermentans*) is a representative organism from this order which is able to metabolize formate (Ergal et al. 2020; Perevalova et al. 2016). The optimum growth is observed between 80 and 82 °C, pH 6.0 (Perevalova et al. 2005). The membrane composition of organisms from this order remains unknown; however, genes for the archaeal lipid biosynthetic pathway (including Tes) have been found in the genome (Perevalova et al. 2005). This archaeon harbors some unique quinone species whose structures have not yet been resolved (THURL et al. 1986). The genome contains the predicted GGR (Desfe_0234) and one paralog (Desfe_0100) (Anna A. Perevalova et al. 2016). The AlphaFold2 prediction of Desfe_0100 clusters with the *S. acidocaldarius* GGR (Fig. 4). The enriched model of this paralog shows a Phe187 residue dividing a potential catalytic cavity into two (Fig. 10b). Cavity 1 is in the close vicinity of FAD (Fig. 10b). The characteristic PxxYxWxFP motif is conserved in Desfe_0234 corresponding to the GGR catalytic cavity (Fig. 6). Interestingly, none of the motifs associated with FAD interaction are conserved in these proteins and the genomic locus does not have ferredoxins or iron–sulfur cluster-binding proteins in the neighborhood (Figs. 6 and 10).

## Thermococcales

Organisms from this order comprise marine hyperthermophiles which thrive at high temperatures and high salt conditions. *Thermococcus kodakarensis* is a model organism from this order. The membrane of this organism consists of ~ 50% DGDs and ~ 50% DGTs at the start of the stationary phase (Gagen et al. 2016). Twelve hours after the start of the stationary phase, the membrane is dominated by GDGT lipids which constitute ~ 75% of the membrane (Gagen et al. 2016). Trace amounts of unsaturated GDGTs have also been reported in the membrane (Bauersachs et al. 2015). *T. kodakarensis* is assumed to regulate its membrane fluidity by altering the length of hydrocarbon chains of the membrane phospholipids (Matsuno et al. 2009). Apart from membrane phospholipids, apolar polyisoprenoids corresponding to lycopene are found in minor fractions in the *T. hydrothermalis* and *T. barophilus* lipidome (Salvador-Castell et al. 2019). In *T. hydrothermalis*, four acyclic tetraterpenoid hydrocarbons in their di-saturated and tri-saturated forms were identified (Lattuati et al. 1998). The *T. barophilus* membrane contains polyunsaturated species of C_30_ squalane and C_35_, C_40_ lycopene (Cario et al. 2015). The relative abundance of C_40:4_ squalene increases slightly at low temperature and high hydrostatic pressure (Lattuati et al. 1998). Meanwhile, the levels of C_40:2_ squalene increase at high temperature and C_40:3_ decrease at low temperature (Cario et al. 2015). The exact localization of squalene and lycopene in membranes remains unclear. However, a study using neutron diffraction has shown that squalene resides in the midplane of a synthetic membrane consisting of 1,2-di-*O*-phytanyl-sn-glycero-3-phosphocholine (DoPhPC) and 1,2-di-*O*-phytanyl-sn-glycero-3-phosphoethanol-amine (DoPhPE) (Salvador-Castell et al. 2021). Lipid unilamellar vesicles (LUVs) consisting of the aforementioned lipids with 1 mol% of squalene had lower permeability to protons but increased permeability to water (Salvador-Castell et al. 2021).

**Fig. 11:**
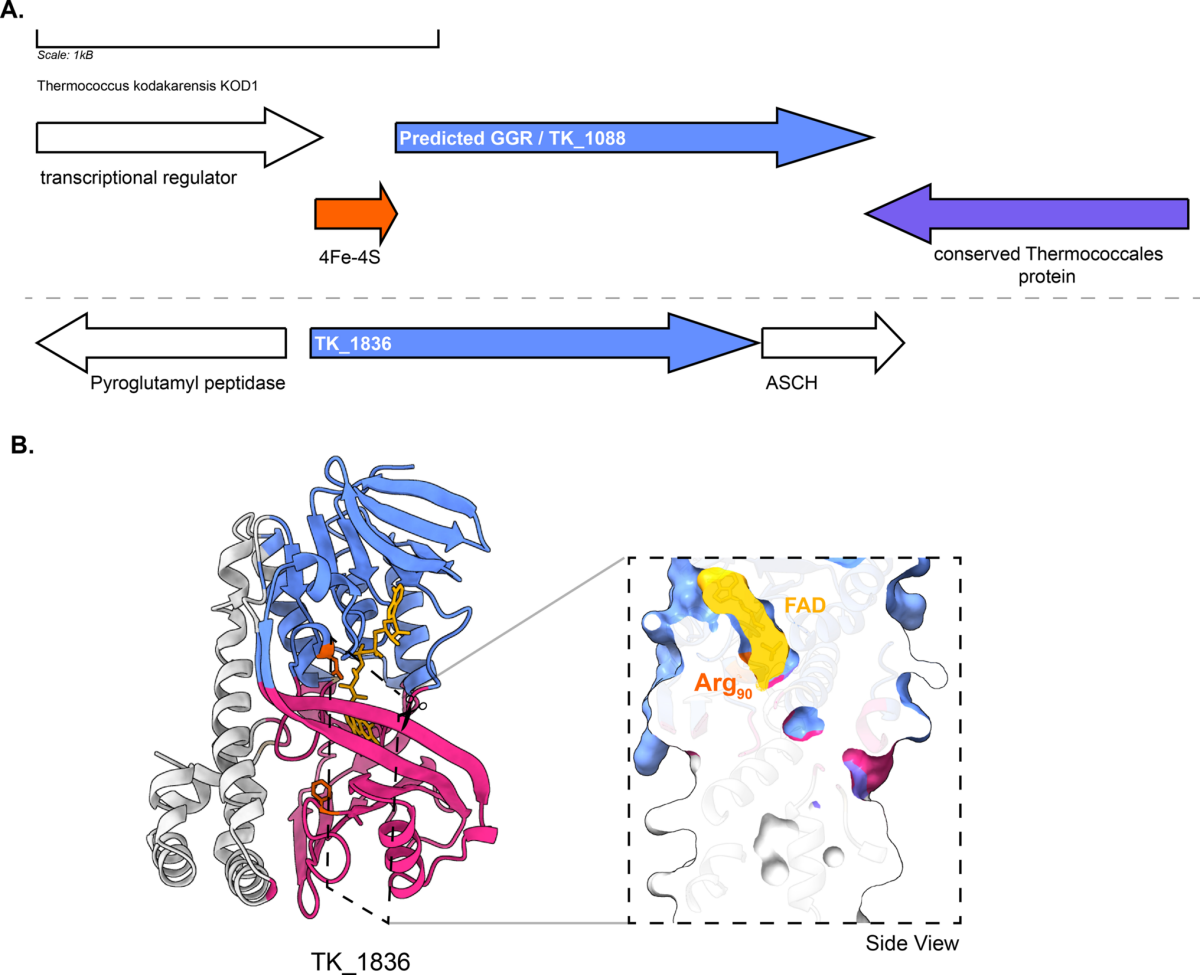
Genomic locus of *T. kodakarensis* GGR and its paralogs, enriched modeled structure of TK_1836: **A** Figure was generated using GeneGraphics (Harrison et al. 2018). *ETF* electron transfer flavoprotein. FAD binding/ETF/ferredoxin-like proteins are colored orange. Conserved proteins in the same order are colored purple. Annotations have been added based on conservation information from the arCOGs or Uniprot. **B** AlphaFold 2 enriched structure and cross-section across the protein surface. The color scheme is the same as in Fig. 2

The predicted GGR (TK1088) from *T. kodakarensis* has 4Fe-4S-binding protein (arCOG00958, conserved in thermophilic methanogenic archaea and other euryarchaeota) upstream in the locus (Fig. 11). The protein sequence comprises the catalytic PxxYxWxFP and the LxGD domains (Fig. 3). The AlphaFold2 structure of this GGR clusters with the *A. fulgidus* and *T. acidophilum* counterparts in the RMSD tree (Fig. 4). All known species of Thermococcales harbor 1 GGR paralog: TK1836 (FixC superfamily) (Bauersachs et al. 2015). TK1836 contains the LxGD motif in its sequence and shares structural similarities with other paralogs from *T. acidophilum* and *Methanoccocus maripaludis* (Figs. 4 and 6).

## Thermoplasmalates

*Thermoplasma acidophilum* was isolated from a self-heating coal refuse pile in 1970 and was classified as a thermoacidophilic organism (Stern et al. 1992). The organism is a facultative anaerobe with an optimum growth temperature at 59 °C and pH of 1–2 (Ruwart & Haug 1975). The membrane is composed of apolar lipids, glycolipids, and glycophospholipids wherein the main constituent is the membrane-spanning DGT (Stern et al. 1992). The quinone species found in the organism include: *cis* and *trans* isomers of thermoplasmaquinone (TPQ-7), MK-7 and unsaturated species of methylthio-1, 4-naphthoquinone (MTK-7) (H. Shimada et al. 2001). The abundance of all quinones is similar under aerobic growth conditions (Shimada et al. 2001). However, TPQ-7 dominates (97%) when the organism is grown anaerobically (Shimada et al. 2001). Apolar polyisoprenoids with C_16_–C_20_ have also been reported in the membrane (Holzer et al. 1979).

**Fig. 12:**
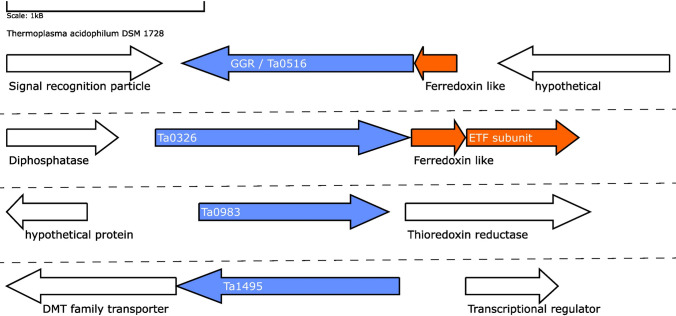
Genomic locus of *T.acidophilum* GGR and its paralogs: Figure was generated using GeneGraphics (Harrison et al. 2018). *ETF* electron transfer flavoprotein, *DMT* drug/metabolite transporter. FAD binding/ETF/ferredoxin-like proteins are colored orange. Annotations have been added based on conservation information from the arCOGs or Uniprot

The GGR from *T. acidophilum* is a membrane-associated and stereospecific as it has a preference for saturation of double bonds in a *syn* manner to DGGGP (Xu et al. 2010; Nishimura and Eguchi 2007). The crystal structure of this GGR was obtained with a bound FAD molecule in extended conformation (Xu et al. 2010) (Fig. 2a). The structure from *T. acidophilum* lacks the amphiphatic α-helices found in the *S. acidocaldarius* enzyme (Nishimura and Eguchi 2006) (Fig. 2a). Enzymatic assays confirmed the essentiality of co-factors like FAD and NADH to catalyze the reduction of DGGGP (Nishimura and Eguchi 2006). The genomic locus of the *T. acidophilum* GGR contains a 4Fe-4S domain protein (arCOG00958, conserved in Euryarchaeota and Thermoproteus); it is likely that this protein fulfills the role of a reducer *in vivo* (Fig. 12).

The genome of *T. acidophilum* contains 3 GGR paralogs: Ta0326, Ta0983, and Ta1495. Ta0326 contains a ferredoxin-like protein (Ta0327) just downstream in its locus corresponding to arCOG01984 (conserved in Euryarchaeotes and organisms from the TACK superphylum) (Fig. 12). Additionally, an ETF β subunit (arCOG00446, conserved in halophiles and TACK superphylum) is present in the locus (Fig. 12). The AlphaFold model of Ta0326 clusters with other archaeal GGR orthologs with either ETF or ferredoxin-encoding genes in their locus (Fig. 5). Conserved motifs associated with FAD interaction (RxxD and LxGD) are found only in Ta0326 and Ta1495 (Fig. 3). Ta0983 does not have any ferredoxin or electron transfer subunits in its locus (Fig. 12). However, Ta0982 in the locus is a protein of unknown function present just in *T.acidophilum* and *T.volcanium* (Fig. 12). The genomic organization and AlphaFold prediction of Ta0983 is similar to the MK specific PR from *A.fulgidus* (Figs. 4 and 12). Thus, this protein could be a putative prenyl reductase for MK.

## Methanococcales

Methanococcales is an order consisting of anaerobic methanogens which can grow in a broad temperature (< 20–88 °C) and pH (4.5–9.8) range (Angelidaki et al. 2011; Thauer et al. 2008). *Methanococcus maripaludis* is a well-studied model organism from this order due to its hydrogenotrophic metabolism which can convert CO_2_ and H_2_ to CH_4_ (Goyal et al. 2016). The organism is mesophilic (optimum: 38 °C) and the membrane of *M. maripaludis* consists of bilayer-forming archaeols and hydroxyarchaeols with galactose or N-acetylglucoamine or serine headgroups (Koga et al. 1998). Monolayer forming GDGTs have not been detected in the lipid extracts from the membrane (Goyal et al. 2016). Apolar polyisoprenoids predominated by C_25_ and C_30_ chains have been reported in the membrane of *M. vannielli* (Holzer et al. 1979). C_18_, C_19_, and C_20_ chains were also found in minor fractions (Holzer et al. 1979).

Thermophilic methanogens, such as *Methanothermoccus okinawensis* (optimum: 65 °C, pH 6.7) and *Methanothermobacter marburgensis* (optimum:65 °C), contain archaeols (with and without cyclopentane rings), isomers of glycerol monoalkyl glycerol tetraether (GMGT), GDGTs, and glycerol trialkyl glycerol tetraether (GTGT) in the membrane (Taubner et al. 2019; Baumann et al. 2022). A switch to glycolipids from phospholipids has been observed under nutrient limitation conditions in *Methanothermobacter thermautotrophicus* (Yoshinaga et al. 2015). Additionally, an increase in polyprenols consisting of 9–11 isoprene units was observed during conditions of energy limitation (H_2_) (Yoshinaga et al. 2015). The production of these phosphate-free polyprenols has been hypothesized to biochemically stabilize membranes (Hartmann and König 1990).

**Fig. 13:**
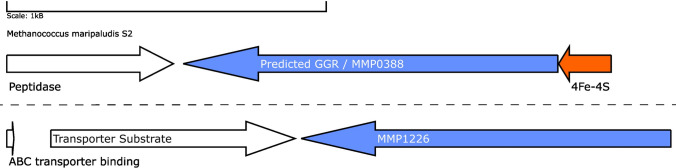
Genomic locus of *M. maripaludis* GGR and its paralogs: Figure was generated using GeneGraphics (Harrison et al. 2018). ETF: electron transfer flavoprotein. FAD binding/ETF/ferredoxin-like proteins are colored in orange. Annotations have been added based on conservation information from the arCOGS or Uniprot

Mesophilic methanogens (such as *M. maripaludis*) have a predicted GGR and harbor one paralog in their genome: MMP1266 (FixC superfamily). Thermophilic methanogens (such as *Methanothermobacter thermoautotrophicus*) have two predicted GGRs (MTH_277 and MTH_1718) and one paralog (MTH_725) in the genome (Goyal et al. 2016; Yoshinaga et al. 2015). The predicted GGR from *M. maripaludis* contains a ferredoxin-encoding gene in its locus and the characteristic PxxYxWxFP catalytic motif; however, it lacks the motifs associated with FAD interaction (Figs. 6 and 13). Interestingly, both the AlphaFold predictions of the GGR (MMP0388) and its paralog (MMP1226) from *M. maripaludis* cluster close to their *T. acidophilum* and *A. fulgidus* counterparts in the RMSD tree (Fig. 4). The RxFD and LxGD conserved motifs are present in MMP1226 (Fig. 6).

**Fig. 14:**
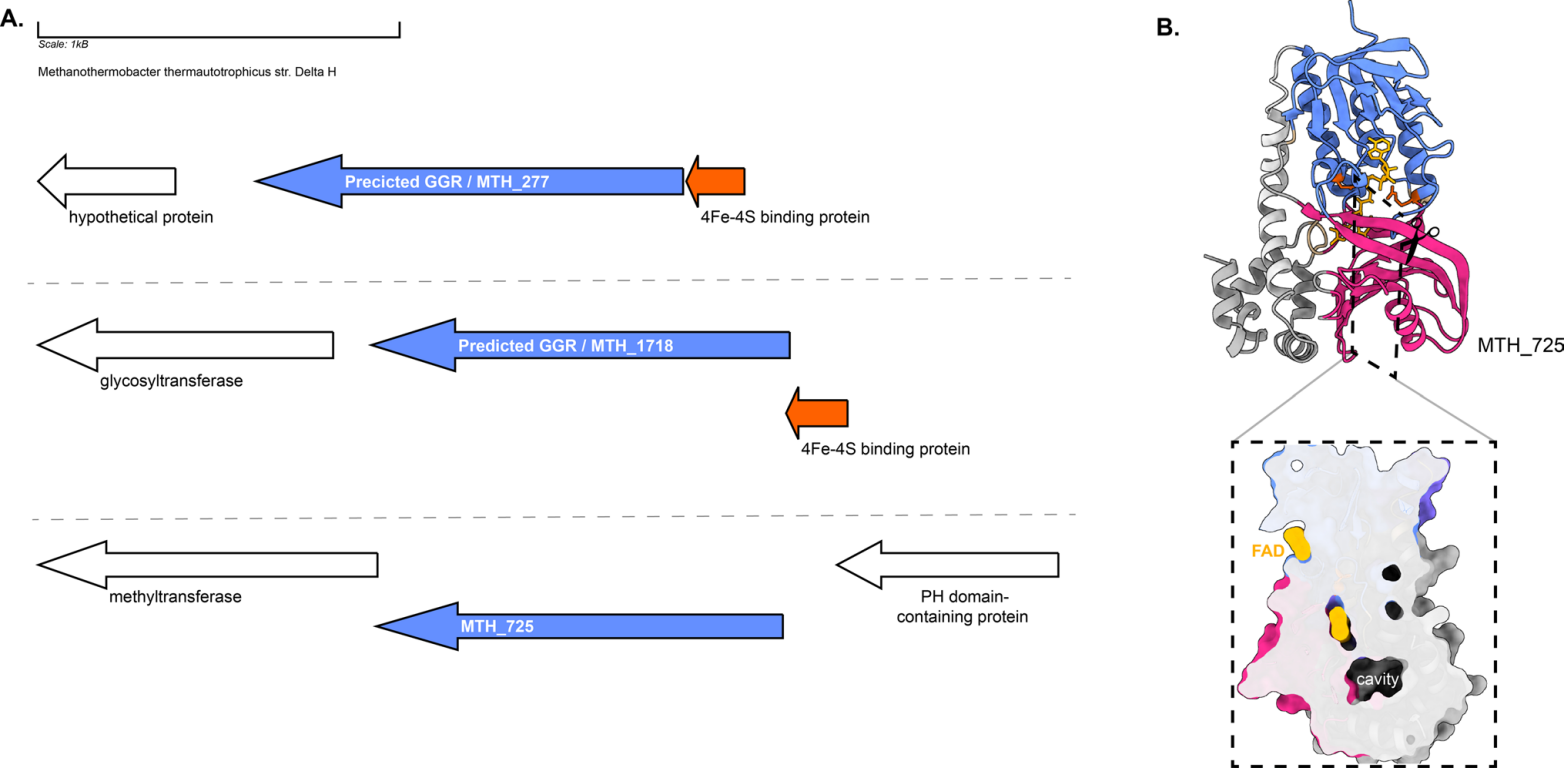
Genomic locus of *M. thermautotrophicus* GGR and its paralogs, enriched model of MTH_725. **A** Figure was generated using GeneGraphics (Harrison et al. 2018). ETF: electron transfer flavoprotein. FAD binding/ETF/ferredoxin-like proteins are colored orange. Annotations have been added based on conservation information from the arCOGS or Uniprot. **B** Enriched structure and cross-section across the protein surface. The color scheme is same as in Fig. 2

Both MTH_277 and MTH_1718 contain a 4Fe-4S-binding protein in their locus (Fig. 14a). MTH_277 and MTH_1718 are structurally similar to GGRs from *M. acetivorans*, *T. acidophilum*, and *A. fulgidus* (Fig. 4). Both these proteins have all the FAD-associated motifs conserved in their sequence (Fig. 6). However, only MTH_1718 contains the catalytic PxxYxWxFP cavity associated with GGRs (Fig. 6). The paralog MTH_725 shares some structural conservation with AF_1023 and contains FAD-associated motifs just like the latter (Figs. 4 and 6). A cross-section across the surface of the enriched MTH_725 model shows a cavity in close vicinity of the FAD molecule (Fig. 14b).

## Concluding remarks

**Fig. 15:**
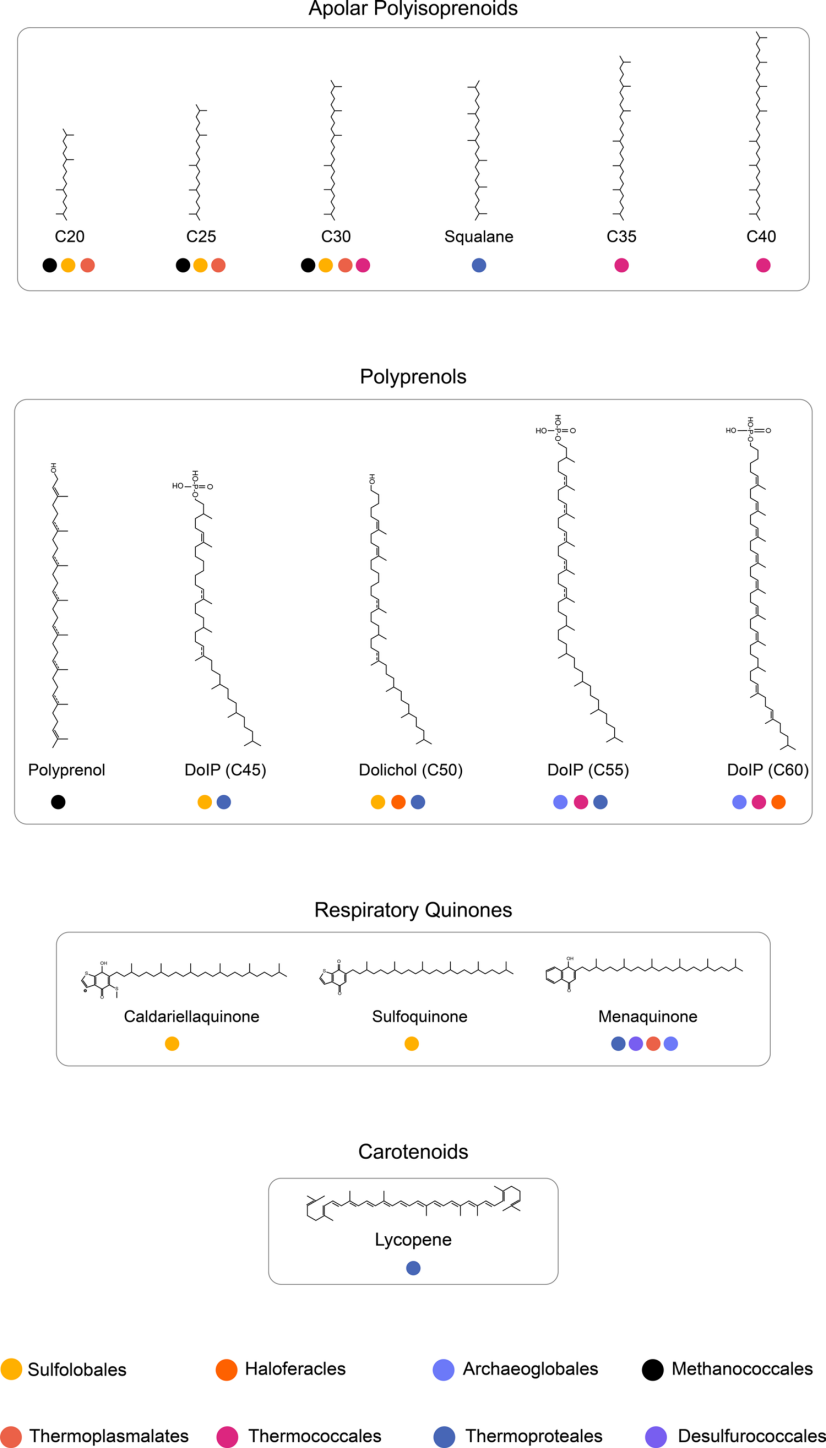
Polyterpenes found in extremophilic archaea: apolar polyisoprenoids, polyprenols, respiratory quinones, and carotenoids discussed in this study. Fully saturated species have been draw for simplicity in most cases. Their distribution among archaea is based on (Salvador-Castell et al. 2019)

There have been considerable advancements in elucidating the biosynthetic pathways of membrane phospholipids in archaea. However, the role and biosynthetic pathways of the various polyterpenes in archaea remain to be investigated. The majority of these polyterpenes have been hypothesized to function in a similar fashion as their bacterial counterparts as there is a paucity of information about their exact localization and impact on the membrane. GGR is an enzyme that plays a key role in the saturation of the isoprenoid chains of the phospholipids; however, this group of proteins is also involved in the biosynthesis of other lipophilic compounds. The saturation states of polyterpenes (Fig. 15) have been correlated with the multiplicity of GGRs in archaeal genomes which are represented by the arCOG00570.

GGRs are promiscuous enzymes and the recent studies have shown that the chain length of the substrate seems to be a regulatory factor for catalysis (Cervinka et al. 2021) (Yoshida et al. 2018). Therefore, it is possible that archaeal GGR paralogs fulfill this void and act on polyterpenes with longer chain lengths for saturation, such as quinones, polyprenols, apolar polyisoprenoids, and carotenoids. None of the GGR paralogs listed in this study contain a signal peptide for secretion, thereby suggesting a cytosolic localization. Preliminary bioinformatics analyses into enriched structural models and sequence alignments combined with extant information from literature reveal that these GGR paralogs are quite diverse structurally and likely functionally. Transcriptomic and proteomic datasets of *S. acidocaldarius* and *H. volcanii* indicate that these GGR paralogs are well-expressed proteins under standard laboratory conditions (Cohen et al. 2016) (Schulze et al. 2020). Interestingly, some of these paralogs along with a few archaeal GGRs co-localize in clusters together with genes encoding ferredoxin or electron transfer proteins that may function as reducing agents and add to the electron transfer. Future study of these proteins should reveal their exact functions as well as their interactions. This should also involve genetic studies to address whether GGRs are essential proteins.

## Data Availability

All data supporting the findings of this study are available within the paper and its Supplementary Information.

## Abbreviations

GGR: Geranylgeranylreductase
G1P: Glycerol-1-phosphate
G3P: Glycerol-3-phosphate
IPP: Isopentenyl pyrophosphate
DMAPP: Dimethylallyl pyrophosphate
GGPP: Geranylgeranyl pyrophosphate
GGPPS: GGPP synthase
GGGP: Geranylgeranyl glycerol phosphate
GGGPS: GGGP synthase
DGGGP: Digeranylgeranyl glyceryl phosphate
DGGGPS: DGGGP synthase
AA: Archaetidic acid
DGD: Dialkyl glycerol diether
AG: Archaetidylglycerol
CDP-archaeol: Cytidine diphosphate archaeol
GDGT: Glycerol dialkyl glycerol tetraethers
GDNT: Glycerol dialkyl nonnitol tetraether
GMGT: Glycerol monoalkyl glycerol tetreather
GTGT: Glycerol trialkyl glycerol tetrather
AF_PR: *Archaeoglobus fulgidus* Prenyl reductase
MK: Menaquinone
CQ: Caldariellaquinone
DoIP: Dolichol phosphate
SQ: Sulfoquinone
FPP: Farnesyl pyrophosphate
